# Activating transcription factor 3-activated long noncoding RNA forkhead box P4-antisense RNA 1 aggravates colorectal cancer progression by regulating microRNA-423-5p/nucleus accumbens associated 1 axis

**DOI:** 10.1080/21655979.2021.2023798

**Published:** 2022-01-16

**Authors:** Zhouyang Cheng, Song Jiang, Ran Tao, Haipeng Ge, Jun Qin

**Affiliations:** Department of General Surgery, Affiliated Hospital of Nantong University, Nantong, JS, China

**Keywords:** Colorectal cancer, ATF3, lncRNA FOXP4-AS1, miR-423-5p/NACC1 axis

## Abstract

Long noncoding RNAs (lncRNAs) have vital roles in the progression of colorectal cancer (CRC). Forkhead box P4-antisense RNA 1 (FOXP4-AS1) showed a potential unfavorable prognostic factor for CRC, while its underlying mechanism remains elusive. Thus, the goal of this research is to determine mechanism of FOXP4-AS1 in CRC occurrence and development. Herein, a Dual-luciferase reporter assay was performed to assess the regulation of miR-423-5p to nucleus accumbens-associated protein 1 (NACC1) and activating transcription factor 3 (ATF3) to FOXP4-AS1 promoter. Hematoxylin-eosin (H&E) staining was performed to detect the pathological changes of tumor tissues. Flow cytometry, cell counting kit 8, Transwell, and wound healing assays were conducted to assess apoptosis, proliferation, migration, and invasion of CRC cells, respectively. The results showed that FOXP4-AS1 was highly expressed in CRC cell lines and tissues. CRC progression was promoted by the overexpression of FOXP4-AS1 in HTC116 cells and animal models. Furthermore, FOXP4-AS1 served as a molecular sponge for miR-423-5p, and NACC1 is a direct target of miR-423-5p. MiR-423-5p silencing or overexpression of NACC1 increased proliferation, migration, and invasion of HCT116 cells while suppressing apoptosis. We also found that the upregulation of FOXP4-AS1 was activated by ATF3 in CRC cells. Collectively, our results demonstrated that ATF3-activated FOXP4-AS1 aggravates CRC progression by regulating miR-423-5p/NACC1 axis, indicating a new target for CRC treatment.

## Graphical Abstract

**Figure:**
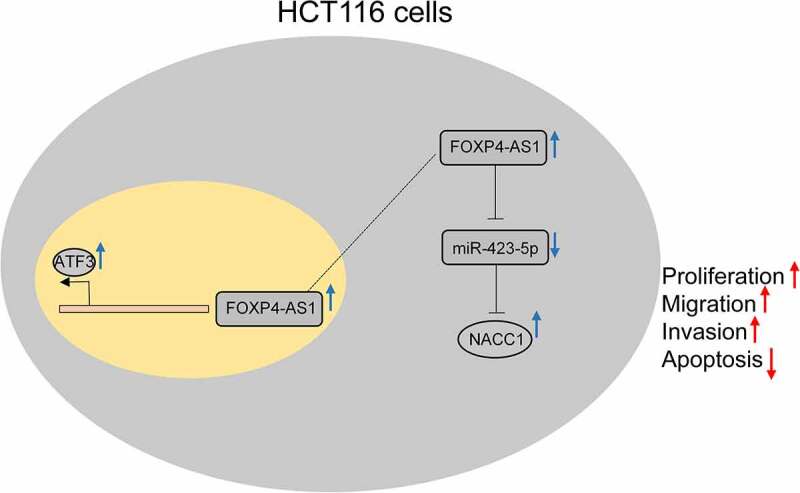

## Introduction

Colorectal cancer (CRC) is currently one of the malignant tumors with a high incidence worldwide. It has an insidious onset, nonspecific early symptoms, a poor prognosis for advanced patients, and a gradual increase in mortality, posing a considerable threat to human health [1]. The diagnosis and treatment of patients with colorectal cancer have advanced significantly during the last two decades. Comprehensive treatment for CRC is based on surgery, supplemented by radiotherapy, chemotherapy, and targeted therapy [2]. However, the recurrence and mortality of patients with CRC have not been significantly improved. Therefore, studies on the development and progression of colorectal cancer and its potential molecular mechanism have tremendous significance for the treatment of CRC.

The occurrence of CRC is affected by many factors such as diet, lifestyle, and genetic characteristics and is the outcome of the interaction of genetic and environmental variables. Long non-coding RNA (lncRNA) exceeds 200 nucleotides in length and is incapable of encoding proteins [3]. LncRNAs participate in various biological functions such as proliferation and differentiation of cells, ontogeny, signal transduction, stem cell maintenance, transcriptional regulation, and post-translational modification [4,5]. Compared with genes encoding proteins, lncRNAs have more substantial temporal and spatial specificity and are closely related to the occurrence and development of diseases. Increasing evidence has indicated the significance of lncRNAs in the development of CRC [6]. As Wang X et al. revealed, small nucleolar RNA host gene 6 is upregulated in CRC tissues and promotes CRC progression by inducing epithelial-mesenchymal transition process and activating the TGF-beta/Smad signaling pathway [7]. Ni W et al. showed that downregulation of growth-arrest-specific transcript 5 by YTH N6-methyladenosine RNA binding protein 3 inhibited proliferation, invasion, and migration of CRC by phosphorylating Yes1 associated transcriptional regulator [8]. In addition, several lncRNAs were identified as biomarkers for the diagnosis and prognosis of CRC, such as small nucleolar RNA host gene 11, urothelial carcinoma associated 1, and prostate cancer associated transcript 1 [9–12]. These finds suggest that lncRNAs are involved in CRC progression by regulating various biological processes. The regulatory mechanisms of other lncRNAs in CRC, however, are elusive. Moreover, lncRNAs have been identified to serve as the competitive endogenous RNAs (ceRNAs) in CRC by binding with microRNAs (miRNAs) to stabilize mRNAs [13–15].

Previous study by Ghasemi T et al. observed the differentially expressed lncRNAs in CRC samples by analyzing three microarray datasets in silico [16]. FOXP4-AS1 has high log fold changes among these lncRNAs. Only two lncRNAs, colorectal neoplasia differentially expressed (CRNDE) and urothelial cancer-associated 1 (UCA1), have higher fold changes than FOXP4-AS1. However, CRNDE and UCA1 were widely studied in CRC. To study them in CRC may lose the novelty. Moreover, we are interested in FOXP4-AS1 since it is an antisense RNA of FOXP4. Our future study will focus on the relationship between FOXP4-AS1 and FOXP4 in CRC. In addition, two studies revealed that FOXP4-AS1 is an unfavorable prognostic factor for CRC [17,18]. However, the underlying mechanism of FOXP4-AS1 in CRC remains largely unknown. We hypothesized that FOXP4-AS1 exerts its function in CRC by the ceRNA pathway. The aim of this research is to investigate the underlying ceRNA mechanism of FOXP4-AS1 in CRC, which could lead to a new treatment target for CRC.

## Materials and methods

### Patient and tissue samples

Thirty-eight paired CRC tissues and adjacent tissues (with a 5-cm distance from the tumor margin) were obtained from thirty-eight patients diagnosed with CRC at the Affiliated Hospital of Nantong University. All specimens were collected during surgical resection. Patients had not undergone radiotherapy or chemotherapy. Among the 38 CRC patients, 22 were males and 16 were females with an average age of 54 years and age range of 29–78 years. Microscopically, 14 were poorly differentiated, 18 were moderately differentiated, and 6 were well differentiated. Eleven patients presented with regional lymph node metastasis. The Ethics Committee approved the present study of the Affiliated Hospital of Nantong University. Each patient was informed and had signed an informed consent form.

### Cell culture and treatment

FHC, a human normal colonic epithelial cell line, and the CRC cell lines HT29, SW620, SW480, LOVO, and HCT116 were obtained from ATCC and cultured in DMEM containing 10% fetal bovine serum (FBS) and 1% penicillin/streptomycin at 37°C in an ambient incubator containing 5% CO_2_.

### Cell transfection and infection

FOXP4-AS1 or nucleus accumbens-associated protein 1 (NACC1) overexpressing or silencing vectors (pcDNA3.1-FOXP4-AS1/NACC1 or sh-FOXP4-AS1/NACC1) with empty pcDNA3.1 vector or sh-NC as the controls were purchased from GenePharma (Shanghai, China). MiR-423-5p mimic or miR-423-5p inhibitor (both from GenePharma) were used for enhancing or silencing endogenous miR-423-5p expression in cancer cells. In 6-well plates, HCT116 cells were seeded and cultured for 12 hours. Using Lipofectamine 3000 transfection reagent (Invitrogen), cells were transfected with FOXP4-AS1, miR-423-5p, and NACC1 according to the manufacturer’s protocols. After 48 hours of transfection, cells were collected for *in vitro* experiments. Sequences of FOXP4-AS1, miR-423-5p, NACC1, sh-FOXP4-AS1, miR-423-5p mimics/inhibitor, and sh-NACC1 were provided in Supplementary file 1.

The lentivirus vector (pGLVU6/GFP) expressing FOXP4-AS1 or sh-FOXP4-AS1 were packaged by Genepharma with the empty vector as the control. HCT116 cells were infected with these lentivirus vectors (multiplicity of infection: 3) for 4 h and then the medium was replaced according to the manufacturer’s protocols. The infected cells were used for animal experiments at 48 h after infection.

### Quantitative real-time polymerase chain reaction (qRT-PCR)

Total RNA was extracted with TRIzol reagent with DNase, and the concentration of RNA was determined using an Agilent 2100 Bioanalyzer (Agilent). Reverse transcription of 500 ng of RNA into cDNA was performed using a miRNA-specific miRNA assay or an mRNA assay kit (Applied Biosystems). Following that, qRT-PCR was conducted using SYBR® Premix Ex TaqTMII on a CFX 96 real-time PCR thermocycler. GAPDH and U6 were utilized as internal controls for the measurement of mRNA and miRNA expression. The 2^−ΔΔCt^ method [19] was used to determine the relative expression of mRNAs and miRNAs. The following conditions were used in the qRT-PCR reaction: 10 minutes at 92°C, followed by 40 cycles at 92°C for 10 seconds and 60°C for 1 minute. The primers used during this study were as follows: FOXP4-AS1: F, 5ʹ-GTGAGCTTCTGGGTTCGACA-3ʹ, R: 5ʹ-ATTGAGGGTTAGGGCAGCAC-3ʹ; miR-4999-5p: F: 5ʹ-GTGAAGATCGGACACTACGTG-3ʹ, R: 5ʹ-CTGCCACTTTATGGCCTGTTA-3ʹ; miR-6890-3p: F: 5ʹ-ACAGCAGGCACAGACAGGCAGT-3ʹ, R: 5ʹ-AGCAGCATTGTACAGGGCTATCA-3ʹ; miR-423-5p: F: 5ʹ-TGAGGGGCAGAGCGAGACTTT-3ʹ, R: 5ʹ-GTGCAGGGTCCGAGGTGGGCAGAGCGAGACTTT-3ʹ; NACC1: F: 5ʹ-CCACCCCAGCGTTTACC-3ʹ, R: 5ʹ-GGGGABCACCTTCCTTTC-3ʹ; ATF3: F: 5ʹ-TTTGCTAACCTGACGCCCTT-3ʹ, R: 5ʹ-TGACTGATTCCAGCGCAGAG-3ʹ; U6: F: 5ʹ-CTCGCTTCGGCAGCACA-3ʹ, 5ʹ-AACGCTTCACGAATTTGCGT-3ʹ.

### Western blot analysis

HCT116 cells or CRC tissues were lysed using 100 mL of radioimmunoprecipitation assay lysis buffer (Solarbio), and protein concentrations were determined using a bicinchoninic acid kit (Boster). Using 10% polyacrylamide gel electrophoresis, the proteins were separated and transferred to a polyvinylidene difluoride membrane (Sigma-Aldrich). Following blocking with 5% bovine serum albumin for one hour, the membrane was incubated with primary antibodies against NACC1 (1:300, ab29047, Abcam), activating-transcription-factor 3 (ATF3; 1:1000, ab254268, Abcam), p63 (1:1000, ab124762, Abcam), B cell leukemia/lymphoma 2 (Bcl-2; 1:1000, ab32124, Abcam), Bcl2 associated X (BAX; 1:1000, ab32503, Abcam), cyclin dependent kinase 2 (CDK2; 1:2000, ab32147, Abcam), Cyclin A1 (1:1000, ab270940, Abcam), and glyceraldehyde-3-phosphate dehydrogenase (GAPDH; 1:10,000, ab8245, Abcam). After three washes in Tris-buffered saline Tween-20, the membranes were treated with secondary anti-rabbit antibody (1:2000, Abcam) for one hour. Gel Imager (Bio-Rad) was used to visualize the immunocomplexes on the membrane using enhanced chemiluminescence reagent. The gray value intensity of the protein bands was analyzed using the ImageJ software. The uncropped gels of Western blotting bands were provided in Supplementary figure 1.

**Figure 1.**
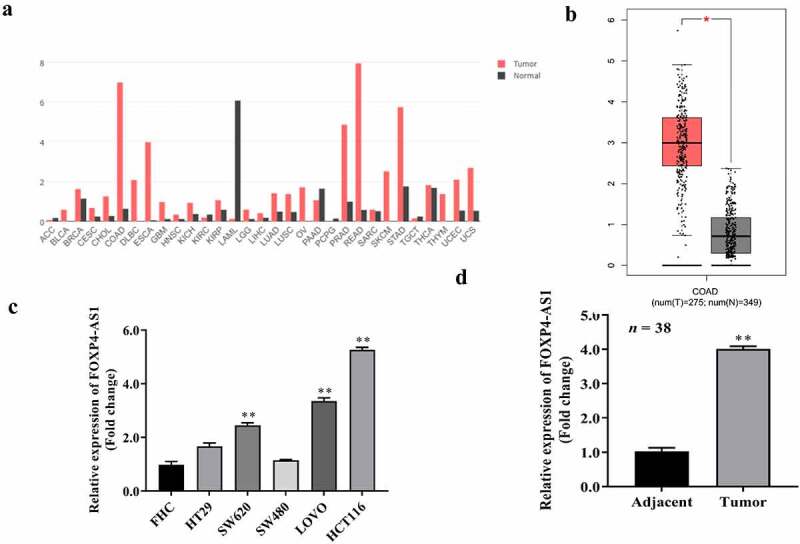
**FOXP4-AS1 was highly expressed in CRC cell lines and tissues**. A, FOXP4-AS1 expression in different cancers according to the expression profile from GEPIA database; B, FOXP4-AS1 expression in 275 CRC tumor tissues based on the expression profile from GEPIA database; C, The expression of FOXP4-AS1 in the CRC lines HT29, SW620, SW480, LOVO, HCT116 was significantly increased than that in control FHC cell line, which was determined by qRT-PCR, **P < 0.01, Vs. FHC cell line; D, FOXP4-AS1 expression in 38 CRC tissues collected in the present study was determined by qRT-PCR, **P < 0.01, Vs. Adjacent group.

### Wound healing migration and Transwell invasion assays

A wound healing assay was performed to detect cell migration. During the wound healing assay, cells were cultured and treated with mitomycin C (10 g/mL) until they reached 80–90% confluence. After 48 hours, cell migration was determined by counting the number of cells that entered the acellular region formed by a sterile insert. For invasion test, Matrigel-coated chambers (BD Biosciences) with 8 μm pores were utilized. In a serum-free medium, 2 × 10^5^ cells were seeded into the upper chambers coated with Matrigel. The transwell’s lower chamber was filled with culture media containing 10% FBS. After culture for 48 hours at 37°C, non-invaded cells on the top of the transwell were scraped away with a cotton swab. Additionally, cells were fixed with 10% formalin and stained with 0.1% crystal violet before being imaged using a light microscope.

### Cell counting kit-8 (CCK-8) assay and apoptosis assay

The CCK-8 test was used to determine cell proliferation. 1 × 10^3^ cells were seeded into 96-well plates and cultivated for 24, 48, and 72 hours. The CCK-8 solution was then added to a 96-well plate and incubated for additional two hours. At 450 nm, the absorbance was determined. Flow cytometry was used to detect apoptosis using an Annexin V-FITC and propidium iodide (PI) apoptosis detection kit (Becton Dickinson). We collected 1 × 10^6^ cells and suspended them in Annexin-binding buffer. Following that, cells were incubated for 15 minutes at room temperature in the dark with Annexin V-FITC and PI and immediately analyzed using the flow cytometry software equipped in the machine (BD FACSVerse; Becton-Dickinson and Company).

### Cell cycle analysis

At 48 h of transfection, cells were harvested, fixed with 70% precooled ethanol at 4°C overnight, and stained with 10 µg/ml PI in the dark for half an hour at 37°C. Flow cytometry was performed on the BD FACSCalibur Flow Cytometer system. Cell cycle distribution was analyzed using FlowJo software (version 7.6) and ModFit LT software (version 3.2).

### Bioinformatics analysis

LncBase (https://carolina.imis.athena-innovation.gr/) was adopted for predicting the miRNAs that binding to lncRNA FOXP4-AS1. The binding site between miR-423-5p and FOXP4-AS1 was also obtained from LncBase. TargetScan (http://www.targetscan.org/vert_71/) was used to predict the targets of miR-423-5p. The binding site of miR-423-5p on NACC1 3ʹUTR was also obtained from TargetScan. The PROMO database (https://alggen.lsi.upc.es/cgi-bin/promo_v3/) and JASPAR database (https://jaspar.genereg.net/) was used to explore the transcriptional factors that can potentially bind with FOXP4-AS1 promoter.

### Dual-luciferase assay

The wild-type, and mutant fragments of lncRNA FOXP4-AS1 and 3ʹ-untranslated regions (3ʹ-UTRs) of NACC1 containing the binding sites of miR-423-5p were cloned into the pmirGLO vector to construct the pmirGLO-FOXP4-AS1-Wt/Mut or pmirGLO-NACC1-Wt/Mut vectors. For luciferase assay, 1 × 10^6^ cells were seeded in 24-well plates and cultured for 24 h. Following that, using Lipofectamine 3000, the pmirGLO vectors and miR-423-5p mimics were co-transfected into HCT116 cells. After 48 hours, the luciferase activities were determined using the Dual-Luciferase kit (Promega).

### Chromatin immunoprecipitation (ChIP) PCR assay

CRC cells were crosslinked, lysed, and treated with protease inhibitors. The nuclei were isolated by nuclear separation buffer and cracked by ultrasound. 100 μL supernatant was treated with 60 μL Protein A Agarose/Salmon Sperm DNA containing 900 μL ChIP Dilution Buffer and 20 μL PI for one hour at 4°C. The supernatant was extracted and incubated overnight with 1 μL ATF3 antibody (Abcam). Rabbit IgG acts as a control. The precipitate was then washed consecutively for 10 minutes with 1 mL low, high salt buffer, LiCl solution, and TE solution. 5 M NaCl was added to de-crosslink the DNA, and it was extracted. qRT-PCR was used to quantify the DNA promoter of lncRNA FOXP4-AS1 in the complex. The primer sequence of the lncRNA FOXP4-AS1 promoter region was as follows: F: 5ʹ-GTGAGCTTCTGGGTTCGACA-3ʹ, R: 5ʹ-ATTGAGGGTTAGGGCAGCAC-3ʹ.

### Xenograft in nude mice

Animal experiments were approved by the Institutional Animal Care and Use Committee at the Affiliated Hospital of Nantong University. Eighteen BALB/c nude mice weighing 17–20 g and aged 4–6 weeks were obtained from Vital River Laboratory Co., LTD (Beijing, China) and maintained in a pathogen-free environment. 2 × 10^7^ lentivirus infected cells in serum-free DMEM were subcutaneously injected into nude mice that were assigned into the control, Over-FOXP4-AS1, Sh-FOXP4-AS1 groups. At 7, 14, 21, and 28-days following injection, the mice were euthanized by carbon dioxide (100% CO_2_ gas replacement rate at 10–30% container volume/min), and the tumors were removed, photographed, and measured with a vernier caliper. The volume of the tumor was determined using the formula (a × b [2])/2, where a denoted length and b denoted width. Tissues from tumors were fixed in 4% paraformaldehyde, embedded, and sectioned into 4 μm sections. The sections were then stained with hematoxylin and eosin (H&E) for 5 minutes.

#### Ki-67 immunohistochemistry and terminal deoxynucleotidyl transferase-mediated UTP end-labeling (TUNEL) analysis

For Ki-67 immunohistochemistry, the sections were incubated with antibodies against Ki-67 (1:200, Abcam) at 4°C overnight. Next, the section was washed three times and incubated with the secondary antibody goat anti-rabbit IgG (H + L). For TUNEL assays, the sections were treated with 0.1% Triton X-100 in 0.1% sodium citrate, and then the sections were incubated for 2 minutes. The section was treated with 50 µL TUNEL reaction mixture and was incubated for 1 hour at 37°C in the dark. Nuclear was stained with 1 mg/mL DAPI, and the images were taken using a fluorescence microscope (Leica, Germany).

## Statistical analysis

SPSS 21.0 software (IBM Corp., Armonk, New York, USA) was used to analyze the data. The mean and standard deviation of the measurement data were calculated. Multiple group comparisons were conducted using one-way or two-way analysis of variance (ANOVA). The t-test was performed to compare two groups. P < 0.05 was considered significant statistically.

## Results

### FOXP4-AS1 is highly expressed in CRC cell lines and tissues

Several studies have observed overexpression of FOXP4-AS1 in CRC tissues than adjacent tissues, suggesting FOXP4-AS1 as an unfavorable prognostic factor. We first evaluated the expression of FOXP4-AS1 in CRC. According to the GEPIA database, lncRNA FOXP4-AS1 was widely expressed in many cancer tissues as compared to normal tissues except adrenocortical carcinoma, kidney renal clear cell carcinoma, acute myeloid leukemia, pheochromocytoma and paraganglioma, and testicular germ cell tumors (Figure 1a). Moreover, the expression of lncRNA FOXP4-AS1 was also dramatically enhanced in the CRC tumor tissues (n = 275) than that in normal tissues (n = 349), as predicted from GEPIA database (Figure 1b). Furthermore, we found that FOXP4-AS1expression in the CRC lines HT29, SW620, SW480, LOVO, HCT116 was markedly higher than that in the control FHC cell line (Figure 1c). Additionally, we observed that FOXP4-AS1 expression was considerably higher in 38 CRC tissues than in 38 matched adjacent tissues (Figure 1d). This suggests that lncRNA FOXP4-AS1 might promote CRC progression.

### Overexpression of lncRNA FOXP4-AS1 promotes proliferation, migration, and invasion while inhibiting apoptosis of HCT116 cells

**Figure 2.**
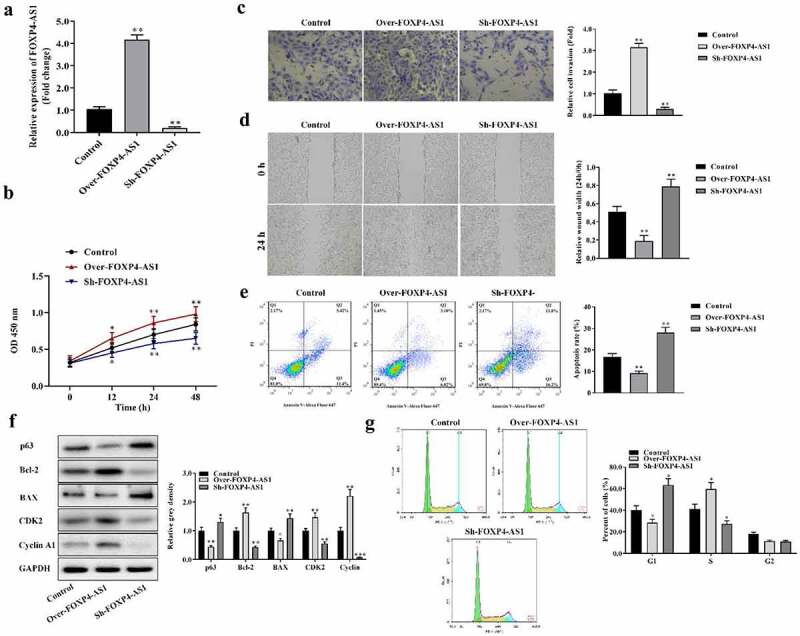
**Overexpression of FOXP4-AS1 promoted proliferation, migration, and invasion while inhibiting apoptosis of HCT116 cells**. A, FOXP4-AS1 was overexpressed or knocked down in HCT116 cells; B, CCK-8 assay was utilized to analyze the proliferation of HCT116 cells under the condition of FOXP4-AS1 overexpression or silencing; C and D, Transwell assay and wound healing assay were performed to detect the invasion and migration of HCT116 cells after transfection with pcDNA3.1-FOXP4-AS1 or sh-FOXP4-AS1, respectively; E, The apoptosis of HCT116 cells was detected by flow cytometry analysis; F, The protein expression of p63, Bcl-2, BAX, CDK2, and Cyclin A1 in HCT116 cells was revealed by Western blotting; G, Analysis on cell cycle distribution was performed using flow cytometry. Data were presented as mean ± SD, *P < 0.05, **P < 0.01, Over-FOXP4-AS1 or Sh-FOXP4-AS1 Vs. Control group.

Effects of FOXP4-AS1 on the proliferation, migration, invasion, and apoptosis of HCT116 cells were evaluated. FOXP4-AS1 overexpressing or silencing vectors were successfully transfected into HCT116 cells (Figure 2a). CCK-8 assay showed that overexpressing lncRNA FOXP4-AS1 promoted HCT116 cell proliferation while suppressing lncRNA FOXP4-AS1 inhibited proliferation of HCT116 cells compared with the control group (Figure 2b). Transwell assay and wound healing assay indicated that overexpression of lncRNA FOXP4-AS1 promoted invasion and migration of HCT116 cells while suppression of lncRNA FOXP4-AS1 inhibited invasion and migration of HCT116 cells compared with the control group (Figure 2c and d). In addition, we found that the apoptosis of HCT116 cells was inhibited significantly in the Over-FOXP4-AS1 group while increased in the sh-FOXP4-AS1 group (Figure 2e). FOXP4-AS1 overexpression decreased p63 and BAX protein expression and increased Bcl-2, CDK2, and Cyclin A1 protein expression. Silencing of FOXP4-AS1 exerts an opposite effect on these proteins (Figure 2f). Moreover, FOXP4-AS1 decreased percentage of cells in the G1 phase and increased percentage of cells in the S phase, while silencing of FOXP4-AS1 exerts the opposite effect (Figure 2g).

### lncRNA FOXP4-AS1 is a molecular sponge for miR-423-5p

**Figure 3.**
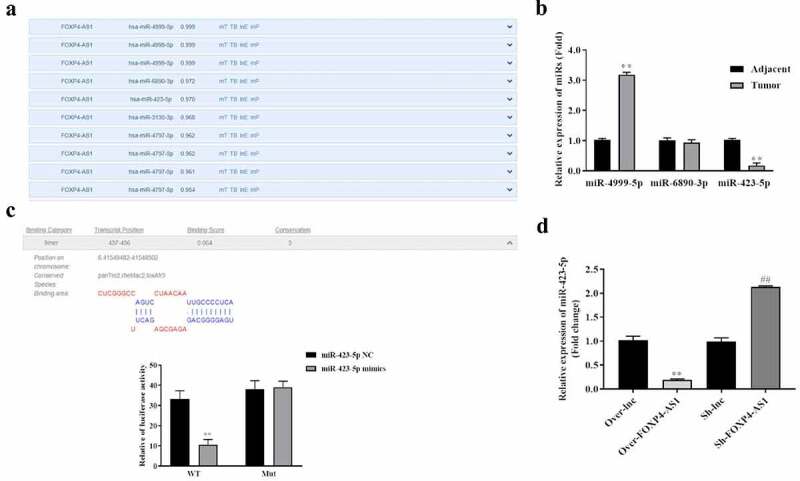
**FOXP4-AS1 was a molecular sponge for miR-423-5p**. A, DIANA database (https://carolina.imis.athena-innovation.gr/) was used to explore the miRNAs binding to FOXP4-AS1; B, qRT-PCR was conducted to detect miRNA expressions in 38 paired CRC tissues and adjacent tissues, **P < 0.01, Vs. Adjacent group; C, Dual-luciferase reporter system was used to detect the regulation of miR-423-5p to FOXP4-AS1, **P < 0.01, Vs. miR-423-5p NC group; D, The expression of miR-423-5p in HCT116 cells after transfection with pcDNA3.1-FOXP4-AS1 or sh-FOXP4-AS1 was detected by qRT-PCR. Data was presented as mean ± SD, **P < 0.01, Vs. Over-lnc group; ^##^P < 0.01, Vs. Sh-lnc group.

To conform the ceRNA hypothesis in CRC cells, the miRNAs downstream FOXP4-AS1 were searched. To explore the miRNAs binding to FOXP4-AS1, the DIANA database was used. The results showed that miR-4999-5p, miR-6890-3p, and miR-423-5p showed the highest scores to bind with lncRNA FOXP4-AS1 (Figure 3a). Further analysis showed that miR-423-5p showed the most significant downregulation in 38 CRC tissues compared with the 38 matched adjacent tissues (Figure 3b). Therefore, miR-423-5p was selected for further study. Binding site of miR-423-5p on FOXP4-AS1 was predicted from LncBase. Furthermore, the dual-luciferase reporter system indicated that the relative luciferase activity of pmirGLO-FOXP4-AS1-Wt was lower in the miR-423-5p mimics group than miR-423-5p NC group. However, miR-423-5p mimics caused no significant changes in the relative luciferase activity of pmirGLO-FOXP4-AS1-Mut (Figure 3c). In addition, we found that the expression of miR-423-5p was significantly downregulated by treatment of FOXP4-AS1 overexpressing vector while upregulated by treatment of FOXP4-AS1 silencing vector (Figure 3d).

### FOXP4-AS1-mediated miR-423-5p promotes proliferation, migration, and invasion while inhibiting apoptosis of HCT116 cells

**Figure 4.**
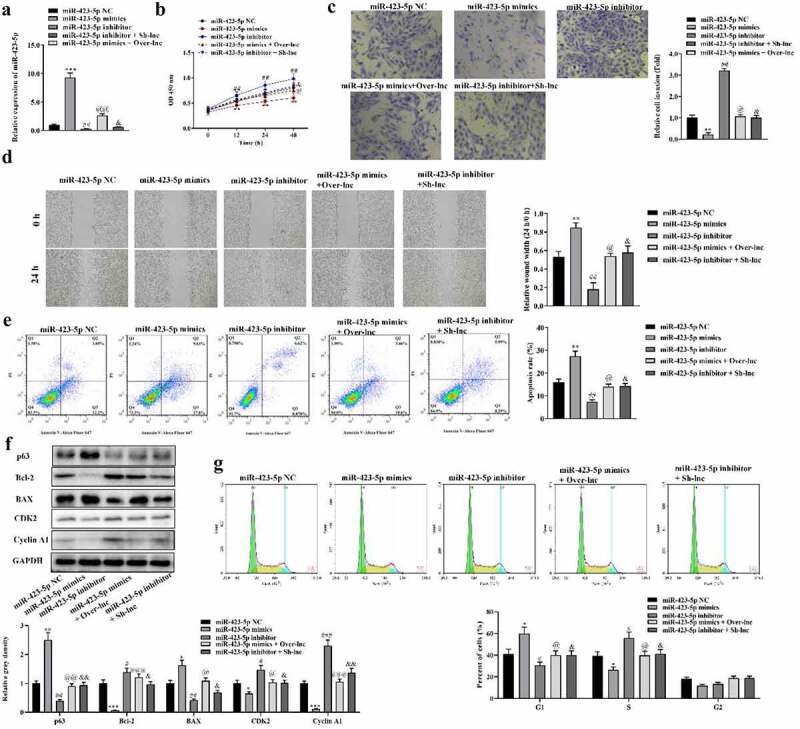
**Knockdown of miR-423-5p promoted proliferation, migration, and invasion while inhibiting apoptosis of HCT116 cells**. A, The expression of miR-423-5p in HCT116 cells after transfections was detected by qRT-PCR. B, CCK-8 assay was performed to analyze effects of miR-423-5p and FOXP4-AS1 on HCT116 cell proliferation; C and D, Transwell assay and wound healing assay was performed to detect the HCT116 cell invasion and migration, respectively; E, The effects of miR-423-5p and FOXP4-AS1 on apoptosis of HCT116 cells was detected by flow cytometry; F, The protein expression of p63, Bcl-2, BAX, CDK2, and Cyclin A1 in HCT116 cells was revealed by Western blotting; G, Analysis on cell cycle distribution was performed using flow cytometry. Data was presented as mean ± SD, *P < 0.05, **P < 0.01, ***P < 0.001, miR-423-5p mimics Vs. miR-423-5p NC group; ^#^P < 0.05, ^##^P < 0.01, miR-423-5p inhibitor Vs. miR-423-5 NC group; ^@^P < 0.05, ^@@^P < 0.01, ^@@@^P < 0.001 miR-423-5p mimics + Over-lnc Vs. miR-423-5p mimics group; ^&^P < 0.05, ^&&^P < 0.01, miR-423-5p inhibitor + Sh-lnc Vs. miR-423-5p inhibitor group.

To reveal the function of the FOXP4-AS1/miR-423-5p axis in CRC, rescue experiments were conducted subsequently. MiR-423-5p expression was increased by miR-423-5p mimics and decreased by miR-423-5p inhibitor, which was rescued by pcDNA3.1-FOXP4-AS1 and sh-FOXP4-AS1, respectively (Figure 4a). CCK-8 assay showed that the proliferation of HCT116 cells was reduced by miR-423-5p mimics and promoted by miR-423-5p inhibitor, compared with the miR-423-5p NC control group (Figure 4b). Transwell and wound healing assay indicated that miR-423-5p mimics inhibited invasion and migration of HCT116 cells while miR-423-5p inhibitor promoted invasion and migration of HCT116 cells (Figure 4c and d). Moreover, flow cytometry showed that number of apoptotic cells significantly increased by miR-423-5p mimics and decreased by miR-423-5p inhibitor (Figure 4e). MiR-423-5p mimics promoted p63 and BAX protein expression and suppressed Bcl-2, CDK2, and Cyclin A1 protein expression. Adverse changes on these proteins were caused by miR-423-5p inhibitor (Figure 4f). Furthermore, miR-423-5p mimics increased percentage of cells in the G1 phase and decreased percentage of cells in the S phase, while miR-423-5p inhibitor caused the adverse changes on cell distribution (Figure 4g). In addition, we found that the function of miR-423-5p on the proliferation, invasion, migration, apoptosis, and cell cycle arrest of HCT116 cells could be reversed by FOXP4-AS1.

### NACC1 is a direct target of miR-423-5p

**Figure 5.**
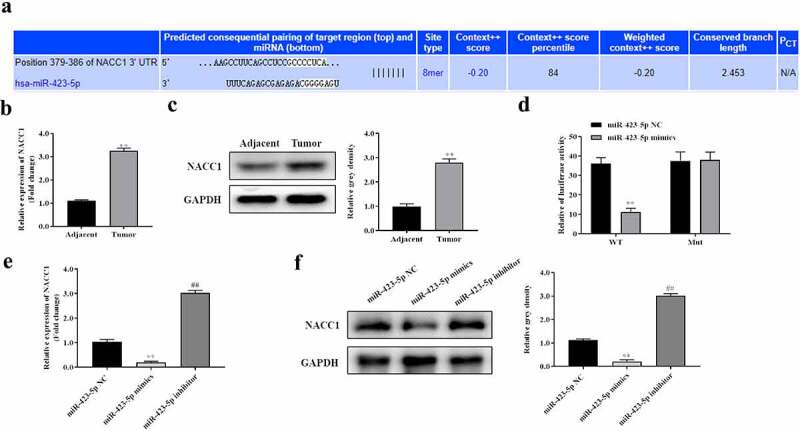
**NACC1 was a direct target of miR-423-5p**. A, Targetscan was used to predict the targets of miR-423-5p and the binding site between NACC1 3ʹUTR and miR-423-5p; B and C, qRT-PCR analysis and Western blot analysis were used to detect the expression of NACC1 in 38 paired CRC tissues and adjacent nontumor tissues, **P < 0.01, Vs. Adjacent group; D, Dual-luciferase reporter system analysis was used to detect the biding effect of miR-423-5p to NACC1 3ʹUTR, **P < 0.01, Vs. miR-423-5p NC group; E and F, The expression of NACC1 was detected by qRT-PCR and Western blot. Data were presented as mean ± SD, **P < 0.01, miR-423-5p mimics Vs. miR-423-5p NC group; ^##^P < 0.01, miR-423-5p inhibitor Vs. miR-423-5p NC group.

Targets of miR-423-5p in CRC were explored. TargetScan was used to predict the targets of miR-423-5p. The results showed that NACC1 is one of the targets of miR-423-5p, which has the binding site at the position 379–386 of NACC1 3ʹUTR, suggesting that miR-423-5p could bind to NACC1 (Figure 5a). qRT-PCR analysis and Western blot analysis showed that the expression of NACC1 was significantly upregulated in 38 CRC tumor tissues compared with 38 matched adjacent tissues (Figure 5b and c). Dual-luciferase reporter system analysis revealed that the relative luciferase activity of pmirGLO- NACC1-Wt was significantly decreased in the miR-423-5p mimics group compared with the miR-423-5p NC group. However, no apparent changes were observed in the mutant group (Figure 5d). In addition, we found that the expression of NACC1 was significantly decreased in the miR-423-5p mimics group and increased in the miR-423-5p inhibitor group (Figure 5e and f).

### MiR-423-5p-controlled NACC1 promotes proliferation, migration, and invasion while inhibiting apoptosis of HCT116 cells

**Figure 6.**
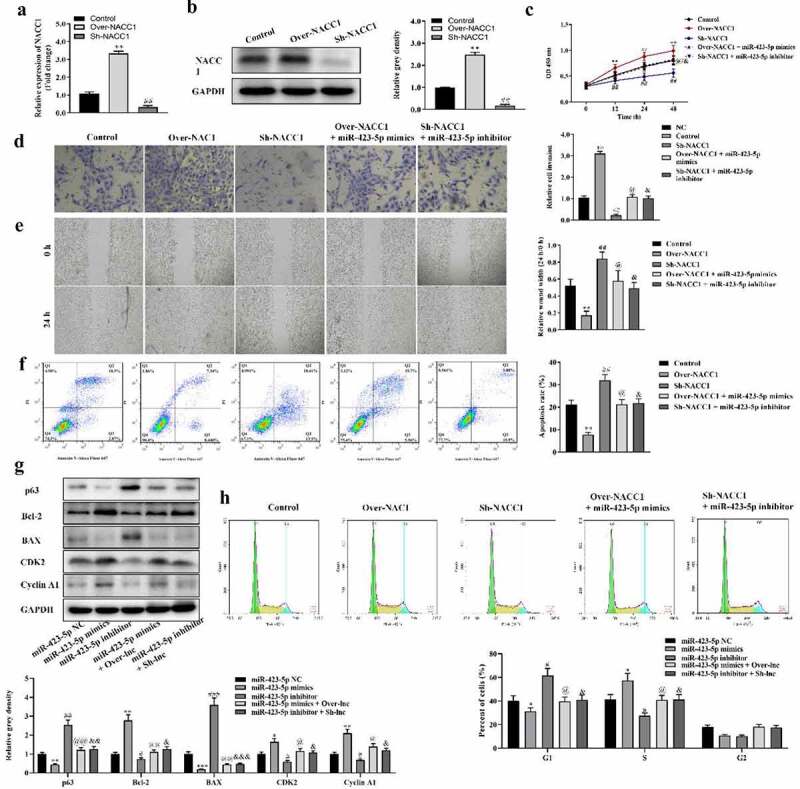
**Overexpressing NACC1 promoted proliferation, migration, and invasion while inhibiting apoptosis of HCT116 cells regulated by miR-423-5p**. A and B, The expression of NACC1 was successfully overexpressed or suppressed by pcDNA3.1-NACC1 and Sh-NACC1 plasmids, as detected by qRT-PCR and Western blot, respectively; C, CCK-8 analysis was used to detect the effects of NACC1 and miR-423-5p on proliferation of HCT116 cells; D and E, Transwell assay and wound healing assay were used to detect the invasion and migration of HCT116 cells, respectively; F, The apoptosis of HCT116 cells was detected by flow cytometry; G, The protein expression of p63, Bcl-2, BAX, CDK2, and Cyclin A1 in HCT116 cells was revealed by Western blotting; H, Analysis on cell cycle distribution was performed using flow cytometry. Data was presented as mean ± SD, *P < 0.05, **P < 0.01, ***P < 0.001, Over-NACC1 Vs. Control group; ^#^P < 0.05, ^##^P < 0.01, ^###^P < 0.001, Sh-NACC1 Vs. Control group; ^@^P < 0.05, ^@@^P < 0.01, Over-NACC1 + miR-423-5p mimics Vs. Over-NACC1 group; ^&^P < 0.05, ^&&^P < 0.01, ^&&&^P < 0.001, Sh-NACC1 + miR-423-5p inhibitor Vs. Sh-NACC1 group.

Functions of NACC1 in HCT116 cells were investigated. The expression of NACC1 was successfully overexpressed or suppressed by pcDNA3.1-NACC1 and Sh-NACC1 plasmids, respectively (Figure 6a and b). CCK-8 assay showed that overexpressing NACC1 promoted the proliferation of HCT116 cells while suppressing NACC1 inhibited HCT116 cell proliferation (Figure 6c). Transwell assay and wound healing assay indicated that overexpression of NACC1 promoted invasion and migration of HCT116 cells while suppression of NACC1 inhibited invasion and migration of HCT116 cells (Figure 6d and e). In addition, we found that the apoptosis of HCT116 cells was significantly inhibited in the Over-NACC1 group while increased in the Sh-NACC1 group (Figure 6f). p63 and BAX protein expression was decreased by NACC1 overexpression and increased by NACC1 knockdown. Bcl-2, CDK2, and Cyclin A1 protein expression was increased by NACC1 overexpression and decreased by NACC1 inhibition. (Figure 6g). Moreover, NACC1 decreased percentage of cells in the G1 phase and increased percentage of cells in the S phase, while silencing of NACC1 exerts the opposite effect (Figure 6h). However, the function of NACC1 on the HCT116 cells could be partially reversed by miR-423-5p, suggesting that miR-423-5p exerts its function in HCT116 cells by NACC1.

### *FOXP4-AS1 promotes CRC progression* in vivo

**Figure 7.**
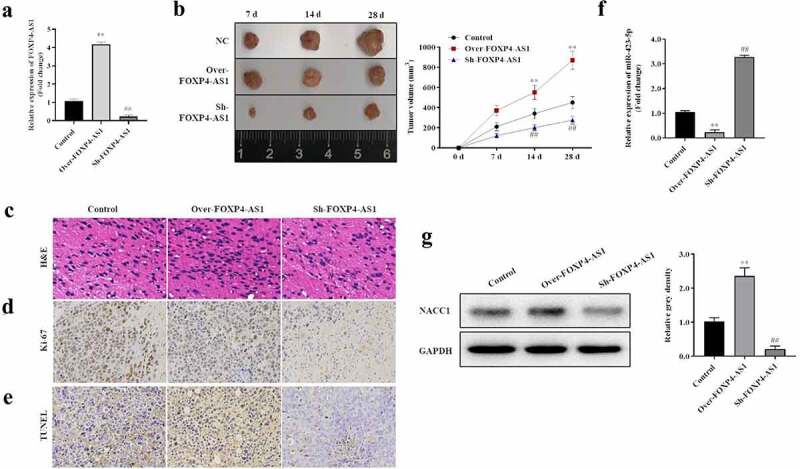
**FOXP4-AS1 promoted CRC tumor growth and metastasis *in vivo***. A, The expression of FOXP4-AS1 was significantly upregulated or downregulated *in vivo*; B, Tumor volume affected by FOXP4-AS1; C, H&E analysis was used to detect the effects of FOXP4-AS1 on inflammatory cell infiltration in the tumor tissues; D and E, Ki-67 immunohistochemical staining and TUNEL were used to detect the proliferation and apoptosis of tumor cells affected by FOXP4-AS1 *in vivo*; F, The expression of miR-423-5p was detected by qRT-PCR affected by FOXP4-AS1 *in vivo*; G, The expression changes of NACC1 affected by FOXP4-AS1 was detected by Western blot, Data was presented as mean ± SD, **P < 0.01 Over-FOXP4-AS1 Vs. Control group; ^##^P < 0.01, Sh-FOXP4-AS1Vs. Control group.

The function of FOXP4-AS1 on CRC progression in mice also investigated. As shown in Figure 7a, the expression of FOXP4-AS1 was significantly upregulated or downregulated in mice tumors by injection of lentivirus-infected cells expressing FOXP4-AS1 or sh- FOXP4-AS1. Further analysis showed that the tumor volume was significantly larger in FOXP4-AS1 overexpressing group and smaller in FOXP4-AS1 silencing group (Figure 7b). H&E staining assay indicated that overexpressing FOXP4-AS1 increased the inflammatory cell infiltration in the tumor tissue while suppressing FOXP4-AS1 reduced the inflammatory cell infiltration (Figure 7c). Furthermore, we found that overexpression of FOXP4-AS1 significantly elevated tumor cell proliferation while inhibiting apoptosis compared with the NC group (Figure 7d and e). Meanwhile, the opposite results were observed in suppressing overexpressing the FOXP4-AS1 group (Figure 7d and e). In addition, we found that the expression of miR-423-5p was significantly downregulated when overexpressing FOXP4-AS1 but upregulated when suppressing FOXP4-AS1 (Figure 7f). Compared with miR-423-5p, expression of NACC1 showed opposite responses to FOXP4-AS1 (Figure 7g).

### Upregulation of FOXP4-AS1 is activated by ATF3 in CRC cells

**Figure 8.**
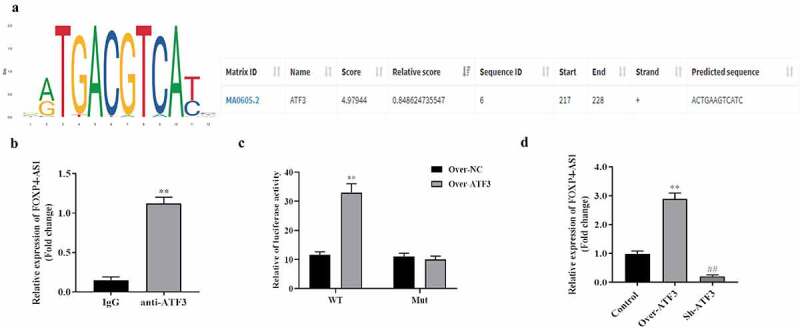
**Upregulation of FOXP4-AS1 is activated by ATF3 in CRC cells**. A, PROMO database (https://alggen.lsi.upc.es/cgi-bin/promo_v3/) and JASPAR database (https://jaspar.genereg.net/) were used to explore binding site between the promotor of FOXP4-AS1 and transcriptional factor ATF3; B, ChIP-PCR assay was used to detect the expression of FOXP4-AS1 in anti-ATF3 group and IgG group, **P < 0.01, anti-ATF3 Vs. anti-IgG group; C, Dual-luciferase reporter system was used to detect the binding of ATF3 to FOXP4-AS1 promoter; D, The effect of ATF3 on expression of FOXP4-AS1 was detected by qRT-PCR, **P < 0.01 Over-ATF3 Vs. Over-NC group; ^##^P < 0.01, Sh-ATF3 Vs. Over-NC group.

Transcription factors play crucial roles in activating gene expression by binding with the promoter of target genes. We explored the transcription factors for FOXP4-AS1 in CRC cells. The PROMO database and JASPAR database were used to explore the transcriptional factors that can potentially bind with FOXP4-AS1 promoter. DNA motif of ATF3 and score of ATF3 to bind to the promotor of FOXP4-AS1 were obtained from JASPAR database (Figure 8a). The ChIP-PCR assay revealed that FOXP4-AS1 was significantly enriched in the anti-ATF3 group compared with the IgG group (Figure 8b). Dual-luciferase reporter system indicated that the luciferase activity of vectors containing wild type FOXP4-AS1 promoter was significantly increased in ATF3 group, while that of vectors containing mutant FOXP4-AS1 promoter presented no significant changes (Figure 8c). In addition, we found that pcDNA3.1-ATF3 markedly increased FOXP4-AS1 expression while sh-ATF3 decreased the expression of FOXP4-AS1 compared with the control group (Figure 8d).

## Discussion

The pathogenesis of CRC is still unclear, and it is currently considered as the result of the long-term effect of environmental factors and genetic characteristics [20]. Several genes play a role in the initiation and progression of colorectal cancer [21]. Therefore, the mechanism of CRC progression needs to be investigated. In the present study, we demonstrated that FOXP4-AS1 was expressed highly in CRC cell lines and tissues, and overexpression of FOXP4-AS1 promoted CRC progression both *in vitro* and *in vivo*. Mechanically, we demonstrated that ATF3 promoted FOXP4-AS1 expression, which exacerbates colorectal cancer progression via the miR-423-5p/NACC1 axis.

LncRNAs are widely involved in critical biological functions in life activities, such as the development and differentiation of individual organisms, reproduction, apoptosis, cell reprogramming, et al., and are closely related to human diseases and CRC [22–24]. FOXP4-AS1 has been identified as a prognostic biomarker for CRC based on bioinformatics analysis [18]. Furthermore, Juan Li *et al*. revealed that FOXP4-AS1 is a functional oncogene in CRC pathogenesis by promoting cancer cell proliferation and suppressing apoptosis. They also generated animal tumor xenografts and revealed that FOXP4-AS1 promotes tumor growth *in vivo* [17]. Based on these studies, we further confirmed the oncogenic role of FOXP4-AS1 in CRC by adding more evidence, for example, it was indicated that FOXP4-AS1 promoted cancer cell migration and invasion. Both FOXP4-AS1 overexpressing and silencing vectors were used for the functional assays, which further confirms our findings. More importantly, the present study innovatively revealed the underlying ceRNA pathway mediated by FOXP4-AS1 in CRC.

The present study found that FOXP4-AS1 is a molecular sponge for miR-423-5p, and NACC1 is a direct target of miR-423-5p. Serum miR-423-5p expression is significantly elevated in patients with stage I–II CRC compared with the control [25]. Inversely, concentration of plasma miR-423-5p is decreased in patients with CRC [26]. Furthermore, Shang et al. revealed miR-423-5p as a contributor in controlling colorectal cancer cell radiosensitivity, indicating miR-423-5p as a molecular candidate for radiation-combined therapy to treat colorectal cancer [27]. We revealed the downregulation of miR-423-5p in 38 CRC tissues. Knockdown of miR-423-5p enhanced proliferation, migration, and invasion while inhibiting the apoptosis of HCT116 cells.

NACC1 has been indicated to contribute to tumor progression in different cancers, such as lung adenocarcinoma, mantle cell lymphoma, and ovarian cancer [28–30]. In this investigation, we found that NACC1 is a target downstream miR-423-5p and is degraded by it. FOXP4-AS1 enhances NACC1 expression by binding with miR-423-5p. NACC1 promotes proliferation, migration and invasion, inhibits apoptosis of HCT116 cells, while these effects were rescued by miR-423-5p, indicating that the tumor-facilitator role of NACC1 in CRC was regulated by miR-423-5p. Furthermore, a previous study revealed that NACC1 transcriptionally activates HOXA9 in CRC cells and regulates CRC cell apoptosis by increasing expression of HOXA9 [31]. HOXA9 is a widely acknowledged oncogene in CRC [32,33]. It can be inferred that NACC1 works in CRC by activating HOXA9.

Transcription factors regulate genes by binding cis-acting elements in their promoter regions. Increasing numbers of transcription factors were found to play essential roles in CRC, such as IRF1, BTF3, and STAT3 [34–36]. FOXP4-AS1 can be transcriptionally activated by paired box 5 [37] and Yin Yang 1 [38]. In the present study, we found that upregulation of FOXP4-AS1 activated by ATF3 in CRC cells. Several studies have indicated that ATFs act as an oncogene in CRC, for example, the induction of ATF3 contributes to anti-cancer activity of Abeliophyllum distichum Nakai in human CRC cells [39]. Yan *et al*. showed that overexpression of ATF3 promotes the pathogenic development of CRC [40]. To sum up, these results demonstrated that ATF3 activated FOXP4-AS1 aggravates the progression of colorectal cancer by regulating the miR-423-5p/ NACC1 axis.

However, some limitations of the present study must be addressed. First, the functions of the miR-423-5p/NACC1 axis *in vivo* need investigation. Second, the limited clinical sample size restricts the conviction of the present study to some degree. Third, the animal experiments lack the evidence to show the effects of FOXP4-AS1 on liver and lung metastasis in CRC. Moreover, there are some other axes that might be regulated by FOXP4-AS1 in CRC by the ceRNA pathway, and more miRNAs controlled by FOXP4-AS1 in CRC progression deserve further studies.

## Conclusion

In summary, we demonstrated that FOXP4-AS1 is highly expressed in CRC cell lines and tissues, and overexpression of FOXP4-AS1 promotes CRC progression both *in vitro* and *in vivo*. Mechanically, we revealed that ATF3-induced FOXP4-AS1 aggravates the progression of CRC by regulating the miR-423-5p/NACC1 axis. These findings provide a solid theoretical foundation for applying FOXP4-AS1 on the diagnosis and treatment of CRC.

## Supplementary Material

Supplemental Material
