# Supplementary material for: Activating transcription factor 3-activated long noncoding RNA forkhead box P4-antisense RNA 1 aggravates colorectal cancer progression by regulating microRNA-423-5p/nucleus accumbens associated 1 axis
Source: Bioengineered. 2022 Jan 16;13(2):2114–29. doi: 10.1080/21655979.2021.2023798 (PMC8973600; doi:10.1080/21655979.2021.2023798)
Supplement: Supplemental Material [file KBIE_A_2023798_SM1492.zip › supplementary/Supplementary file 1.docx]

**FOXP4-AS1 (5’-3’)**

CCCTGGTTTTCTGTGGAAAGTGAGCTTCTGGGTTCGACAGTGGGACCGGCACAGACCTTCCCGCAGCTACAGGCCATACGACAACCCCGCTGCTCTTTCTTTCTGCGGGCACTCGGGCCAGTCCTAACAATTGCCCCTCAAGCTGTGTGTGCTGCCCTAACCCTCAATCCCGAACCCGAAACAGTGCGTGCTGGGACGAATCTAGGCAGGTCTCCGAAACCGGGGTGAAGATGCTGAAGTTCAGGTAAAAACCCGCATTGGCCAAAATGCTCCCGGGCTTTCTCGGGAGTCACCCGAATGGCAACTTCAGGCTCGGGTTTGGTTCTCCGGACTCCCCACCCCTATTCCCGGCTCCAGGCGCCGCGGTCAACCTGCGGGGAGACTAGACGTTGGGGTGCCAGACACGCGGTGATCTCCAGACGCCCGAAACGCCAGGCGCCGAGTGAGCGAGGAAACCAGCCGGGAGGGGGCTGGCCAGGGAAGCCGGGGAGCAAAGGAAAGAGCGGGGCGCGCTCTGGGCTCAGGGTTCCCATACCCTTTCCCCCCAACCTAGGATCGGGGTGGGACGCAGGTGTGTTCACGAGATCGCCCCAGTTGGGGGACACAGGGAATCCGCCGCCGTCTGGGCCTGTCTCAAAATCCGCATTCCGCGAAATTACCCGGTCCTTTATGGGGGCGGCCGGGACCCCAGGTCGCATTCTAGCGCCACTGGCTGTGCTCTGACGCAGCCCAAGGAAGTTTGTTTGGCGTGGATGAGGAGCCCGAGGCTGAAGCGCCGCCGGTCAGAGAGGTGCTGGGGTTGTCCAGGGGAGATACCAGACTCCTCAGTTGGGGCGGGGGGGGGTCCTGCTCGGTCACTCCGGGCTCCTTCCCTGGTGGCCTCCGGGCCCCCGTGGCCGCCCTCTCTTCCCCCCTTAAGTGGTGAAGCGGGAGCTCTCTCCACAGCGGGTGAGTAGAGGTGTCAGCCAGATCGTTTCCCCACCCTCCACCTCCGGCGCACAAATCACAGGGGCCATTTCCAGACGCTCTCAAGTCCCCAAGAAGTTTTCCGCTCTATCCCCGAGCTGACCGAGCGATGGAAAAGGGGCTGACTGGCCCTGGCACTTCCGAGGTGCGACCTCAGCAAGACAAGTCCCCTCTTCACCAGAGACGACACGGCGGGTAGGACCGCGATCCCCCGCTCTCTTGCCCCCATTTTCATCTGTCTCCGGCGCGTCCTGGGGAGTCCGGTACCGACTAGAGTTGGGGGGAGCCACTTCCCTTCCCAGCTCTGGAAAGTCTGGGCCGGGAAGTATTCTTTGTCTGACTCGGGGCGGGGAGGGGGTCGCGCCCCCGTACACCCCATTCCCAGAGGCCCGGCGGGGAGGGGGCGGGGCCCAGATTCTTGGGGAAGGGCGGGCCTCGGTGGCCTCTCCGGCCGCCCCCCTCCCCGCCCCGGCCTCCGGCTGCCCCCACTCCCGCCGCGCCACAGCCCCCACCTGTTCCCGAGGCAGCTCCGGCCCGCTGGGGCCCGGGCCAGGGGCGGAATGTCGCGGCTGCGCGGGGTCAGTGCGCCACGGCCGGCCCTGGCTGGAACAAAGTGCGCCGGCCCGGGCCCGGCTCCTCCTGGCGCCCGGCGCCCAGCGCGTCTCGGGCGGGCAGTTTGCAAACACAAAGTGAGCCAGCGAGCGGGCGAGAGAGTGCGCGAGCGAGGGAGTTGGTCTGGTCGCCCGCCCAGCCCCCACCCACGTCCCTGGGCCCGACCCGCCACCTACCTGTCCCGCCCGGCCGCTCCGTGCGGGCTCCGGCCCGTCAGCCCGCGCGGGGCATGGGGCTCGCTCCGGTCGCAGCCCGGGCCCGCCGCCGCCCGCCGGGGCTGCCCTTCCGCGCCGCCCCGCGTCGCGCAGCTCCCGGGCGTCCTCCCAGCGCGCCGGGCCGACCGCCGCGCGCCTCAACCCCGCCGGGGCATGGCACTTTGTGGGCTGCTTGGCTCGCTCCGGCTCCGCTCTTCCTTCCCGGTCCTTCCGCGACGCTCGTCAGCTCGCTCCGGCTCCCGGGCTCCCGGACCCCGGCGTCCCCGCCCCCGGCCCAGCCCTGGTTCCGGCCCGGGCTCCTCCCCGCCGCGCCGCCGCCGCCTGCGCCGCGCCCTCCTCGCGGGCTGGGGGCTCCGTCCCGGCCGCCGGCCGCCGGCCGCACTGTCCGGTCCGCAGGGACGGCGGGCTCCGGACTCCGAGGGGCTGCGACTGAGCCTGGGCTGCGAGTGGCTGCGAGCGGGCGGCGCGCGGGCGGGGCGGGAGGGGGCGGGGGAGGAGGAGGGTGGAGAGGGAAGGAGGGAGCGCGCGGAGAGCGAGGGAGGAGGGAGCGAGAGAGCAAGCGGCGGAGGGAGGGGAGACCAACTTCTAGTGCTACCATAATTCGCCTAAAGCAGGTGCAGCAACTTTCACCCCGCCTGGCAGCCCGCTGAGAGCGAGAGAAAACCACGTGCACCAAACTTTAGGGGAGGAAAAATGGGAACGTGGGGAGAAGACGTGAGGCGGCAGATCTGAAAAAGATGGTAGGTGGAGGGACCCCAGGGATTCACCCTGGCCTCGGACTAATCCTATGGGGCGCTTGCAAACCCCGGCCATCCCCTGGAGTCTGAAGGTACGGCCAAGTCCTGATCCCACACGGTTCACTGGTTCCCGGCGGGCTGCGCTCGGCGCGGGGAGTGGCCGGGGGCCCAGAGACGGAGCCAGTGTCAGGCCCGGGCCCTGTCTGGCCTCCCTCCTGCTCCCCACTGCGTGGGGGCTGGGGGTGGGCTTCCTGAGCCGAGGGTAGAAGGTGGGGAGGCCCCTTCAGGGCTTTGTGAACGGTCACCCCCTGGCGCGCACTGCCTCCCTCTGCGGAAAGGGAATTTCCCGTAAAGTGCTCTGCGAACCAGTGAAGAAGGGGCCCAAGGCCAGAGCCATGCGGGCAAAGTGAAGTGCAAAGTTGAGGCAATTATCCGGACAAATTAGATTGTAAACAAAACCCCGCGTCTCTGAACATCGCTTTTGCCCAGGGGGACTGGAGGGATTGTGGTGGTGGCGGCGATGGTGTGTTTGGGTTGGGAGGGGGAGGTCTGGGTGAGGAAATGCCCACGGCCCAAGGGGGAGGGCTGTGTTGTTAGCAGACGCACCACTCAGAGTCCCGTGGAAATCTCAATACCCGTCCCCCTCCCCGCCCCACACACGCCGGAAACATCTCCCATCTGTCTGCATGTGTTGGGGGTGGGGAGAAGTGAAGGAGGCCTCAGGGGTCCTCATGAAGTACCCCAACCCAGCTCAGTGATGCCGGGTCCCCATGACCAAGGGAGGATCCTGTTGTGGGATCAGGACAAGAAGACAGTGGCTCTTGAAGCCTCTCCTGGCCCTAAATCCTCAGGCAGGTGGACAAGAGGCTGGCATAGTATGTGAGCCCCTGTGGAGTGGGAGCTGGAAGCCTGAGGTGTGGGGTCGGAGCTGCAAGGATTGATGCTTAGTTGCCTGGGGGGAGGGGATTTCTTGGCCGTTCCAGTGGTCCTTGAAAGAAAAGGGGACCTCAGAGAGGAGGCACCCTCTTGGGTTGTTTTTCATCACTTCTGTCCCTTTTCTGCAATGTTAGTGACACCTGCACAGGTCCACATTTTATGTATTTGCCTTTTTGGGCAGGTGGGACAGGCATACTTAGGTCCTGCTGGAGCGTTTTAGATTTCTAGGTGTGAAATTGTGCAGGGAAGACTCTAAGTGCCATTTATGTGGATATCTCAGTGTCCTTTCCAGTGTGGCAACTGAGATAACCATAGCCATAAGAATAAAATAACAGCTAACATCTGAGTCGTACCACCCACCAGCCATTTAATCTTCACAGTAACCCCAGGAGGGAGATATTTGTATTATCTCCATTTTATAGAAGAGGAAACTGAGGCACAAAGAGATCAAGTAACTTGCCCAGGCTTACACAGCTGCTGGTCAGTGATGAGCCAGATTCCAACTAGAGCCACTTCAGTGAGGACAAGAAAGAAGCTCATGGTGGTCTCTGAAGCGACACTGCCTGCTTGTGGGTTAGCTGAGGGGAAGGATATTCTGGGAGAAAGAAAGCCCTTGGTAGTACTATCTGATTTACCTTCTGTTGAGGCCCAGCTCTGGTGGAAATCATGGTGTGTGGACAGCCCTAGGCCAAGCCTGGAGTGGCAAGTCCTTGTGTCAGACCAGCTGGGCCAGTGACTATGTCCTCAGTTCTCCTTCCTATCCTTCTTGCAGCAAAGGCTGAGGGTGGGGTGAGAACTAGGGGCTTTGGAGACAAAAGAGAGAATTTCTGTAGGGGGGTATTGAATGAGGTGACCTCCGAGGGGCTTCTCCAGCCCTTTTCTGGCTGGTTCACTGAATGTTTTTCCCATCCCCTTCCTGCAGCAGCCCTTGTACCTTACACATGGGCTCACTTGCCCAGAGGGATTGCCCTGGGAAATGCTTCACTTTCAGTTCAGTTTACCCCTCATCTCCAGGCCAGTTCAGTTCCCAGCTCCCTGTGAGCTACAGTGGGGGCTCAAGGCATCAAAAGACCCTGTCTTGTCTTCACCTAAAGGGCATTTTAGCGAGGAAGACAAAGCCTCCCCAAAGAAAGATACAGAACAAATGCCCCGAAATCAAAGAAGCCAGTCCAAAGCCCTTCTTCCTGGTGCAAGAGCTTCGTTAAGAAGGTTGAAAAGAGTAGCCCTTGGCTCCAAAGTGGGAGTGACCGGGAGCAGGCAGGAGGGTCCTCTTTCTGTCCTTGTTCCTTCACAGGACTCAGGGCAGGTGCAGGTGCTTTGGCGGTGGCCGGAACCAAGGCTCATGGCTTGGCCCCTCCAGGGGGGCCATAGTTAAGAGCACAGGGGCTAGAGTCAGGCAGCCTTGGGTTGAACTTCAGCGTCTGTCACTTAACCAGTGTGTGACCCAGGCAAGTTACATGATGTTTGAGTCTCAGTTAACTCACTCATCACCTCACTCATCAATTGGGAGGTTGGGAGGCCTCCTTGGGTGTTTGGGAGGGAGGATGGGCAAAACTTCTAGCCTGGTAATGTGTTCCTCACATGCTTTCCATAATTACTTATTTTCCCCTTGATGAGCTCCAGGGTCCTCACTTAAAATGACGAATGCTGACCTCACAAAACTGCAAATCATGAGTTGCCACTTATTAAATGCTTGCCAGGCAGTCGAATAACGTGCTGTTCAGCTTATCACCCCAGCAATGGTGACTCTTCTGCAGGTGAAGCTAAGAGAGGTCAAATGACTTGCTCAGTTCACATAGCTAGGAAGTAGTGTGGCGAGGACTTGCTCCCAGGTCTCCTGCTTTGAAGCAGACGCCCTTTCCCAAGGTCAGCCGCCACCCTATTGTGGAGGTGAAGTGGGCTGATGGATGAGCTTGTTCCTAGCAGCAGGCCTGATACTCAGGAGTAGGTTGGGGTGGTAGTGGTGTGTACATCTAGAAACATTTGCTTCATTCCCATGGTCCCAACAAACCTAAGCCCAGACAGTTTATGTTTCTATTATTATTATTATTATTGAGACAGAGTCTCACTCTGTTGTCCAGGCTGGAGTGCAATGGCACAATCTCAGCTCACTGCAACCTCCGCCTTCTGAGTTGAAGCAATTCTCCTGCCTCAGCCTCCCGAGTAGCTGGGACTACAGGCTCATGCCACCATGCCCTGCTAATTTTTGTATTTTTAGTAGAGATGGGGTTTTGCCATATTGGCCAGGCTGGTCTCGAACTCCTGACCTCAAGTGATCTGCCCGCCTTGGCCTCCCAAAGTACTGGGATTACAGGCATGAGCCACTACACCTGGCCAAAAACTATTTTTTAGTTATTGAGGAATAAAACTTCATCAGCATAGGCCAGGTGTGGTGGCTCACGCCTGTAATCCCAGCACTTTGGGAGGCCGAGGCAGGTGGGTGGATTACCTGAGGTTGGGAGTTTGAGACCAGCCTGACCAACATGGAGAAACCCCATCTCTACTAAAAATACAAAATTAGCCAGGTGTGGTGGCACATGCCTGTAATCCCAGCTACTCAGGAGGCTGAGGCAGGAGAATCGCTTGAACATGGGAGGTGGAGCTTGCAGTGAGCTGAGATCGTGCCATTGCACTCTAGCCTGGGCAACAAGAGCGAAACTCTGTCTCAAAAAACAAAAAACAAAAATAAAACCCACTTTATCAGCATAATACAGTAACTCCACTCCACTCCACTCCTAAGTATATGCCAATAAAAATGCATATGTACATATGTTCACCAAAAGGCATACACCAGAATGCTAGGCCATCTACTCGCATGTGCTCACTATTCACAACAGCTGTCAACAATGGAATAGGTGACTAAACCACGGTCTTCTCATCCGTGGGCTAGTCCTTGGCAATGAGAATGAGCAAACCATTGCCACGAGCAACAACATGGAGGAATCTCACAAATCCAACGTTGAGCATAAAAGCCAGACACGAAAGAAAACACCGTTTGATTCCATTGATAGGAAATTCAAAACCCAATCCAATGAATTGATGGCATTAAAACTCAGGAGCATGGCTGTTTTAGGTGGGGGTGTGACGGAGGGGCACAAGGGGATTTCTGGGGGTCTTTTTGAATTTGGGAGTTGGTTACATGCATGTGTTCACTTTGTAAACATTAATTGAGCTATACATTTTATGCTTAAAAGTGAAACATGTAAAAATCATTGCTAATATAGGCCTTTCTGTGACTCTCCTTCTTTCCCATTCTCCATCCTTCCCTTGTGGTTTTCTGAAGTTGGCCTTCCCAAGTAAGTTTTCTACTCTTACTCCTTTGGTATTGCTTGGGTATCTCAAAATTTTCTTTTGTTTTAGTTATTTATTTTTATTCACTTTTGGGGCATAGTAAAGTATGAAGCAATTAAAATTTTAAATGAAAGGTAGCCTGCTCCTTGTTTTTACTTGTGTTTTGCTGAACTTCATGCTGCCGAGATGAATGTCTACATGTTTGTTGCATGTGTTGTGTGTGTCTCTAGGTCACTTACTTAAGAGGCTGCTGGAGAGGATTCTACCATATCAGTGTGTCCCATCTTGTTTTGTTACCCAGCCTCCCACTGATGGGGCAGTTCGATTGTAGTGGATATTTGGGTATTATGGTCTCCTTGCTGCAGTAGGCTTCCTTCTGTGACCTCCCTCCCCACTGTGCCTCTCCTTCCCCCTGTGGTTCTTCTGCTCCATAATCTTTTCCCTTTCCCATCTCCCCACTGTTCCTCCATTCCCTGCTTCCCAATCTTTCTGACTGGGAAAGACCTAGGATCTGTGCCGATTGGAGGTGAGGGAGGGATAGGAGAGGGACAGCCTTTTCCTTTTCCACAAAGGCTCCTCTTCCCCAAGATCAATGATGATTTAAAGAAAGAAATATTTCATTAGGGGTTGACATCTTAATGAACCGTGCCAGGAACCTTCAAGGCACTCTCTAGGCTTCCTCGGTTTTAAGCGGGGCTCCTACCTCTGGGGGAGGCTTATCAGTTTACCATATTGCCCCAAGTAGATGACCATGGTAGTGTGGGTAGCTGTGCCAGGGGCTGTGGAGGAGAGGGCCCCCAGGACTGGGGCGAGGCTGGCTTGTCCTTCTAGGTGCCAGGCTGACCGGGTGCCTGGGCCCTTGGAGGTATTTGGAGTTCCTTCTGGGACGTTTAGGGAGTCAGAGACTCAGGCCTTGTCCAAAAGGTGGTGGGCATGGGACTTTGGGCTGATTGGTGGAAAATCAGTCATTAAAGATCAGGAAATAAGCGATAGCTTGACAAGCAGTGAGCAATTTCCCACTAGTTGGAACACAGCATCTTTAGGTCAAATGAGATCCCTACATGGAGGGATTTCTGTGCAGGAAGAGAGCAGGACACACAGAGAGGGGCCAAGAGAGCAGACCCAGTGCCTTATCCCTCCCCCATGCACCCCACCCATGGGGGTGGGGATGGGGTGGAGGATGCATGAGGGTAGCAGAAGCCCCAGGAGTGGGCACAGAGAAAAGAATTCCTTTTTCCTTCATTTAGTTCTTCCTCATCTTTTTTTTTTTTTTTTTTTTTGGACACGGAGCTTCCGCGTTGTTGCCCAGGCTGGCGTGCAGAGGTGCAATCACAGCTCACTGTGATCAACCTCCTGGGCTCAACTGATCCTCCCACTTCAGCCTTCCGATTAACTGTGACCACAGGTGTGAGCTACCATGCCCGGGTAGAGAAGGGGTCTCTGTGTTGCGCAGGTTCGTCTCAAACTCCTGGACTCAAATAATTCTCCTGCCTCAGCCTCCCAAAGTGCTGGGATTGCAGGTGCAAGCCACCTCGTCCGGCCCTTCCTTATCTTTTAAGAGCTATCCCACATTTTCCATCCACTTGAAGACTTTTGGATTTAAGTCTCAATTCTGTCACTTTTCTAGCCCCAAATCCTGAAGCAAGGGGCTTAACCTTAGTTTACTCAACTGCAAGATGGGAAGAAAAATGAGACTCTGGCAGAGAGGTCGTGAGGACAAGTATGGCTCATGTAGGATTCTTCTCAGTTTAGGCATTGGATAGACTATAGCTACTGTTATTCTTTCTTGATTAACCCAGCCCACTTCTGCCTCTTTCCAGGTGCTCTTTAGCACTCCTATGTCATTGATGTTATAATGTGTTGTTAGCTAACTTATTTAATAAGCTCCCAGTAACTTTTAACAAACCCTATTGTTTGTTAACTGTTTAGCCATTTTATGCCTCATTTCTCCAACTAGATAATAAACTCCTTAATTTAGGGACCCACAATTTCAGTCATTTGGGGATTTCTCATTGCATCCAGCTTAATTTCTTTTGCAAGGTAGCTGCACAGTACATGCATATTGATTTGATTATGATTTGATACTTTAGAGCAAAGTTTAAAAAACAAAAAATAGCCTAACGCTTTAGGGCAAGTTGGTGTCCTGCAGGTATCAGCTGCATGTTGTAGGACATATGTAATAACTGTGTCTTCAGTGATCGCAAGGAAAGAGCTAGCTAGGGTCAAGGGGGAAACCTGGGTAGAAAATGGGACAAGGAAGACTCTCATTCCCTCAGAGCCATCCCCTTCTCTCAGAGGGCTGTGCTCCCCATTCTTCTCATCTCTACTCTGGCTCCCTTTCTGCTTGTTGACTTAGGATGTCTTCAGAACTCTGTGCTTAGCAGAGGATGTATTCCTTCCCCATGGAACCTGGAAGACCTGGGGCTTGGAGTCTCAGCTGCTGGCACTGGAGGGTGAGGTGGGATATGAAGGAGGGAAGTTAAAGCCCAGGTAGACTCACATTGAAAATAGCTGCCACCTGTTGAGCATCAACTGTTCACCAGGCACTTTTAATATGTATCTCTGATTCCCACAACAGTCCTATTTTGTTTTTGAGAGAAAGCAGTGCTCCAAATGTCAGAGGCAATGAATGTAGAGTAGAAATAAGTGAAGATAAGAGTGTCTTTATACATCCCTTATTGTGTGTGGCATCTGGCTGGGTCCATGGAGTGGGGCTCACACACTTCCTCATCCTTGTAATTCTATGCCAGGCCTGGGCCCCAGGCCCCAGCTCCTTCCTGGTTCTCCTGCACCCCCTCTTCTTTCTGGTCTTCACCATGTGCCCTAGGGTCTTCTCTCCTCCAGTCCCCTCTGCTCTTGAGCCATGGCTCTCCAGCCCCCAGGACCTCTCTCAAGGTTGTTAGGCAGCAGGAAGTCCCTGGAGCCAGGCAGCTGAAGGCAAACTGAAGCTGGGTCTTGCTTAAAGAAGAACCACCCATGCATCAGACACTGTGCTAAGCACGCTCCACACATGACCTCATTTCACCTCCCAACAGTCCTCTAAGATGATGCAACAGATACTTGTTGGTGCCCTGTCTGGTGCACTGTGACTTTTCACCCCTAAGGCTTCCAATCACTAGTGCCTGACTTTCCTAGCCTAAGGGTTTCCTTTGGGAATTAGAGCCCACTTTTCCACTGTGTGGGAGACCAGAAGTGCCAGGGAACTAACACTTCCTGGGGGAGCCCTCCACCCATAACTGATGGGAACTGGTATATAAATACCCCTACTCCCTTACTCCCCTTGTGGGATAACTCCGAGTCATGTGTGCCATGCATACTCCCAGGGTTTCCTCTGTGGGACTGAGCCCATCCTGTAGCTGGCTTGGTAACATATACTTTGTTGCCTCCTTTCCTTGTCTTACTTCCCCACTTTTCTACTGGTATTTCCTTCACCTCCTAAATCAACCATTCGCAATGGAATAGTGTCTCAGGGGCTGCTTATGTGGGAACCCAGCCTAAATCAGGTTGATAGTATTATTATCTTCATTCTATAAATGACAAGACTAAAGCTCAGAAAGGTTGAAATTCATACACGGTTCCACAATTATGTAAGTACAGCACCTGTCTTCATACCCTGGCTTGTTACACACCAGAGTGATCACCTCATTGCTCTAGTATACGGCTTCTTGAGGGCCCTTTCCCTCTCAAGAAGACTGGTTCAAGGCGATTCCCCTTGGCCTCCTGCCTTGAGAGGTAGGAGGTGGGGAGCAGGGTGGGTCAGGGGTACACTGGGAGTATTTCCAGCCCACCTAGCCCCCCGTCTCCCTGTGGGACACTGGCAAGCAGTCATAGCCAGTCTCTGGATTGCAGGCAGACAGAGAAGCAGAGAACACCTACGGGGCTGGAGATATGATGAATAGAAAGAAGTGAAAGCCACCTGGAGTGCTGGAAAAGAAGAGTACTGAATCAGGGGTACAAGGCGAGGGCGGGCTCGGGGCTGAGGAATCTTTCTGAGCATTTGCAGTACACAGCAGCTCCTGCAAAAACAGAAGGAATGAGATGAGAGACAGGAAAGTATTTATTTTTTTCTGTCTTATTTTAGAGTCAGGATCTCACTTTGACACCCAGGCTAGAGTGCAGTGACATGACCATAGCTCGCTGCAGCCTTGAATTCCTGGGCTCAAGAGATCCTCCTGCCTTAGCCTCTGAGTAGCTGGGACTACAAAAGGATTTCTTGATGTTAGAGTTTGCTCAGCCAGGGAATGTGGAAAGTCCCCTGCTGAGAACCTTCGGACCAGACAAGAAATTGGCCTCTTTTATGAGAGTGGAGGTGGGCCCCTTGGGACAGGAAAGACCTGATCCCTCCAGAGACAAAAGTTACCTGGTGAGAACACCTTCATGTTTTCTATTTGTTTGGTTTTCATGACATGTTTGTCTTAGTTTAGGTTCTCCCTGAAGCAGACTCTGACAGTGATTCAAGCATAAGTGGTTTATTTGGGAGGTGGTCCCAGAAATACCAGTGGGGGAGCAGGGCAGGGAGAATGAAGGGAAGGCAGCCAATAAAGGGAGTGTTATCCCACTGTCAGCAAGAGGAGTGCAGTCCCACTGAGGACTTGAGCGTGCGTAACACACACCGCACACAAGCCAGCCCACGGACGCAGGCCTTAGGGTACTCATCCACCAACTTCCCCTTCATCGTGGGCTGCGTCCTCCTTCTAGGCGCATTCACTCTCCAGCACTTCCGGCCTGCTCTGGGCTGGAGCCAAGGGTGTTTCCACGGCCAGGAAAAAAGCCTCAGGCAAACAGCAGGTGTTGGCAGTAAGCAGGCTTCTACCTTTAGAGGCGAACACTGAGAGAGATGGGCAGGGCGCCAGTGCCTGCCACACTCTGCCGGTCTGTTCTCCTGCTTAACTTTTCCTTTTCATGAGTCTTTGTGGGCTCATCTCATCTCCCTCTGTCCTGTCTGAAGCACTGGGCGTTGCCCCACTCTGTCCTCGTCATCTGTGCTCTCGGACAAGCCCATCTACTCTCATGGTTTTACCTTCCACTTTTGAGATGGTGGCTCCCACTACTATCCTGTGTGGGACCTGGATCCCATGTGCGTGTTTGTGGGCCTCCAGGGATGGCACACTCAACATGTCCCCAGCCCAAGCCATCTTCTGTATCCATAACATGCTCCACCTCTGCATTTTCTCCCCATTGGTGGCACCATAATCTACCCAGTCACTCTAGAAACTAGAATCATCCCTTGGTTCTCCTTATCCCTTCCTCTTCACATCCAGTCACCAAGTCCTGTGGCTTCCAAGGCATTTCTCAGAGTCATTCCCTCCTCTTTACCTCCACTTCTCCTACTATTGGTCAGGGCAGGACCTTCATCATCCCCCATCTGGAGTATTCCAATAGCTTCCTACCTGGCCTCCCCACTTCCAGCCTTGTCCTCTTCCGGAGCCTCCTTTCAGTGCATCCTCCACAGTGGTGAAAAAGTTCTATCTAACATGCCAGCCCAGGCTGGGTGTGGTAGCTCATGCCTGTAATCTCAGCACTTTGGGAGGCTGAGGCAGATGGATTGCTTGAGGCCAGGAGTTCAAGACCAGCCTGGACGACATGGCAAAACCCCATCTTTAATAAAAATTAAATAAATACAATTCCAGCCTGAACTTGTCATGCCCTGCTTAAGATGCATTCAAAATTCAAGCACCTTGGTTGGGTGCACAGGGTACAAGGTATCCTTTCTTGAACCTCTGGTTCTATCCCTGGCCACTCCCAGCTCTGCCACCTGGGACTTGTGGCATGTGCTATGCTCTTCCATGTAGCTAGGCTTCCTCTCAGACTGTGTCCTCTGTTTGGAATATCTTTTCTCCCATATGCTTTCACTGCTGACTAAGACCCAGCTCAGATGGCACCTCTTCTTGGAAGTCTTCCCTGAATGCCCCAGGCTGGATTAGACACTCTTCTTTTGTGTTCTTGTATCAGTGCAAACATCAGAAAATCCACTTCAAATGGGCTTAAATAACAATGAAAATTGACTGACTCTTGTAACTGAAAGTCAAGGGAGCAGGACTCGCTGCAGATGATGTGTGATCCAGGGGCTTAAAATGATGCCCTTGGGTGCTCCCATTGCTTAACTCTGCCTTTTCCTGGGTGGGCTTTATTCTTAGAGTCCTTGGAGGGAGTCCCATCAGCTTCAGACTCACATCCAGCCCACTTCAGTCCAGTGAATTGTTCAAGCCAACACTTAGCTTATTTGGACTTGACTAGGGATTGACTTGCCCTTCTCCAAACCCATCAAAATGGCCAAGGGCTTACAGTGTTCTACTTCAGGGGTTGGTGGATTATGGCCAAATCCTGCTTACCACTTGTTTCTGGAAATAAAGTTGTATTGGAGCACAGTCATACTCATTTGCTTACGTATCATCTGTGGCTGTTTTTGTGTGACCGGGGCAGAGTTGGGCAGTGGAGGCAAAGACCAGCAAATTTTGCTGTATTTTAACCTCTACAGGCTATGAGGCAAAATTGAGAATATGATGCAGGGATTTACATAACAAGAGAGAAAACAAATTTCCACAAAACTTTTATTGATGAAATTAGAATGACAATAATAATTGAGAACTTTTGTTTGTACTACAGGTTTACTAATAAGAAGAATGAAATTTGGGGAAAGGGGATAACATTTTGCTTGGTTGGGGTTCAAAGTTAACGCTCCCTATTATCAAAGTGACCACCAATGTTCATCTGTGAAAACCACTTAGCTAACAGACCATACAAAACCAGGCAGGATTTTGTCTGTGAGCCACAGTTTGCAGACTCCTGAATTAAATCAAAGCATTACAGAGCTAAGGGAAGAGACAGTGGGAAGCTGATGCTGGAGATGATACTGACCCCATAGGTCTGTGGGAGGCATCTTATAAGGCATTTCTTTATAAGGCAAGTTCATGTCCATTGAGCTCCTTAATCTTGACAACAATTCCATGAGGGTAGGCAGGGTAAGGATGGTTGTACACTCTTCAGAGAGGAGACTGAGGCTCAGAGAGAAGTGGCTTGTCTGAAGTTTCATAGCAAAGCCTCTCTGGGACACCTTTGAGGATGCTAAGACATGGGCCTTGGAACATGTGCTTTGTTGAGTAATTGAACAATTATGGTGTCTCCTGGTTGGAAGACAGGCTGAGCCTGCAGAGGGACAGGCTGGTCTGAACATGGTCTGGAAGGCTAAGTTTCCAGCTGGCTCTGTGACATCCAGTGGTCTCCAGGAATGGGGCTGGTCAGCATGGTGCCTAGGAACCCAGTGTGGCTTCAAGGTTCTCTGAGCATAGGCCTGTCTCCCTATGCAGGACTGGGGATACCAGCATAGCAGCTAGAAAGAAATCAGGGCTGGGCGTGGTGGCTCACATCTGTAATCCCAGCACTTTGGGAGGCCAAGGCAGGAGGATTGGTTGAGACCAGCCTGGGCAACATAACCAGACCCCTTCTCTTTTTCTTTTTTTTTTTTGAGATGGAGTCTCTCTGTGTTGCCCAGGCTGGAGTGCAATGGCGCGATCATGGCTCACTGCAACCTCTACCTCCTAGGCTCAAGCGATTCTCCTTCCTCAGCCTTCCAAGTAGCTGGGATTACAGGCACCCACCATCATGCCCGGCTAATTTTTGTATTTTTGTAGAGACAGGTGTTTTGTAGAGATAGATGTTTCATATTGGCCAGGCTGGTCTTGAACTCCTTACCGCAGGTGATCTGCCCGCTTCGGCTTCCCAGAGTGCTGGGATTACAGGTGTGAGCCACCATGTCTGGCCATGAGACCCCTTCTCTACAAAAAATACAGTAATTAGCCAGGCATGATGGCGTGCGCTTATAGTCCCAGATACTCAGGAGGCTGAGGTAGGAAAATCTCTTGAGCTCAGGAGGCGGAGGTTGTCATGAACCAAGATCTCACCACTGCATTTCAGCGTGGGCAACAGAGCAAGACTCTGTCTCAAAAAACAAACAAAAAAATAGAAATCAGACTTCAGGCAATAATGCCCAGCCCCACAAACAAAGATTTTGACCCTCTACCTCACTGCCTGCAGCCTTCAATGCCTGGCCTCATCTGGGTTCCTGAGGAGCAAGCCTGCATCCTGTGTCCTGTGGGCAGGCTGTGGGCAGCCCTCCTCCAGGCTTACCTTGCCTCAGGTAGGGCTGCCCAAGGTCAGGGAGGGTGGGTGGAGGCAATCAGATGTGAAGGACAGATGCTGAGTTCCTTTCCAGGATGATGATTTGTGGGTACTGAGCATGTGTGTTCTGGTACATACAAATACACAGACACTTCACACATTCCCTGTCTCACACACGTTTTCACACTCCCATAGTCACCTCCATCTTCACTACCACCATCTCATACTCACATACATGCACAGTCTCGTGGTTTCTCACTTTTTTTTCTTTTTTTTTTTGAGATGGAGTCTGGCTCTGTCACCCAGGCTGGAGTGCAGTGGCACGATCTCAGCTCACTGCAACCTCTGCCTCCCTGGTTCTGGTGATTCTCCTGCCTCAGCCTCCCAAGTAGCTGGGATTACAACCATGCACCACCACACCCAGCTAATTTTTGTACTTTTTTAGTAGAGACAGGGTTTCACCATGTTGGCCAGGCTAGTCTCGAACTCCTGACCTCAGGTGATCTGCCCGCCTTGGCCTCCCAAAGTGCTAGGATTACAGGTGTTAGCCACCGCGTCCAGCTGGCTTCTCACATTTTTTCATGCACTCTCAAGCTTGCACAACATATTCATATGCACAACATGTTCATACATACCTCTATTAGTTTCCTGGGACTGCCACAACAAAGTATCACACACTGGGTAGCTTAAAACAACAGGAATTTATTGTATCAGTTTTGGAGGCCAGAAGTCCAAAAGCAAGGTTCCTTCTGAGACTTGTGGGGAAGGGCCTTTCCTTGCCTTTGCTGGCTTCTGGTAGCCCCAGGTATTCTTCGGTTTGGAGGTGCATCACTCCAGCCTCTGCCTCCATCTGTCTCTGTGTCTTCACATGGACATCTTATAAGGACACTAGTCATAATGCATGAGGGCCACTCTATTCCAGTAGGGACTCATCCTAACTGATTACATCTGCAATGATACTGGTTCCAAATAAAATCATATTCGGAGGTTCTGGGGTTAGGACTTCAATATATCTTTTTTGGGGAGGAGCACAATTCAACCCAAAATACCACCTACAGCCATAAAATCTCACATACATAGGCCATACTGCCATAGTGACACAGATATAAAACACACAATCCCATAGTCACAAAAATCACACACTCCCATATACACTTTCAGGATCAGACACTCCCATGGTCCCCCCACATGCACGCATACACACACATGGCGAGGTCATCACATACACACTCCTTTAGTTTCCCAGTCTTACAGCCCTACACACCCCCTCCAGTCACACCCCTTCGCACACCCTCCTGCCCCCGCCTCACTCCCAGAGGCCACCCTGTGGAGGTCATGCTCAGAGACCCGGCACATTTGCAGCCTCGCACTTGGCTCATACATCTCACATCCTCTCCCACCCACACAGTCTCTCCCCTCTGACTCCTCCGCCCAGCTCACCAGTTCCCGCACCAACCCTCATGGCAAGGAGGCAACAGACTGGCTCTGTTCCACTCCAGCGGGGGCACCAGGTGGCTCCCCCCTTGTCCTCTGGAATCTGATGCCCCCACAAGCCTGCCTTTTCAGGCTCCCACGCTTCCCTCTCAGGGACAGACAGACAGAAACATCCACACACTCTCATGCTCACACACGCGCCCCTCCGGCAGCCCCACGGCAGCAGTCTGTTCCTCATTATGGGCCAATGCGCCTGTTTACTCACCCAGCTGGAGATGGTTAGAGAGTGATAAATAATGAAAGAGAAAACAGGCTTTTCCCCTCTGTGGCTTCTATTTATGTAATATGTGTCACTGTGCATTCACAGACTTTGTTTCGGAGCTGGTTGCAGGAAATAGTTTATTTTTTCCGGACGGTGGCTTAAGGGGTGGGGAGGTGGGGGGCAGGGTTTGGGTTTCTGATCTCACGCCCATAGGAGTTTCGGAATCTGGCTCCCAGAGTCCTCTTCCCCTGGTGCCTCCTCTCCTCGCCTCCCCTGACATCCCCGGGGTGGGCATCATGGCTAGAGGCTGCCTCAGAACCAGGTTCTCCCACTCCACCTATTTGGAATGGGCTGGGGTGGTTGGGGGCCCCTCCCAGCTGCCTCTGAAACCTCCCAGACAGATGGGCTTGTTTTAAGAGCCAGACAGCTTGAGGCTGGACTCCCAGAAAGGAGGCAGTTGGACAGGGTCTTCCTAGGTAGCCTGCACCTGTTGTTGGGGGAGGATGCAAAAGGATTAATTCTATAAAATAAGAAAAAAAAAGATCCATACCTCATGTTCACTTAGCACTTTCATCCCCGTGGTCTCTCTGAGGCCTCACAACTTTTAATGGCTGCTTCCCAGGTGAGAGAACTGGAGGAGGGCTTGCCATGGTCTGTGCAACTGGCTGATGAGGAGCCAGAACTTCACACTCAGGCCTCTGAGTCCAGAAGAGCCTCACACACCCCACTGCCTCCTCCTCCCAAGGATGCCTCCCTTGGTAAAGTTCTGGTTGCCCTCACTCATTCTAAGGTGATGGGAGTAGTTATAAGGGTGTGGGGGTTGGAATTTGGGGAATGGTGAGTCATCAGAGGTCTGAGAAGTGTCCCCTTCTCCACCCTTTCCTAAACATTAAGGTGAGCCTCCTTTGGGTGACAAGCCAGGGAAGGAAGAAGCAGCAGAAGGCTGCCCTGCTCCCCAACCCCCAGTGCAGGGAGGCTCTTGGGAAGCTGGAAGGGCACCTGCCCCCACCTGAGCCTGTCTTTTTGAGTGCCAGGGCAGGGAGACAATGTCTTTGTTTGACATGGAGCCCTGGTTGTATCCCAGGCCACCTGCTGGGGGGACCCAGAGGTTCTGACCTTTGAGGAATATAGCATCCACTATGGAAAATGGGCCCAGAAGCCCCCAACTCCTGCAGACTGTGGGGGATGAATATATACAGGTGCCATGGGATTAAGGTGCAGGGCAGAATCCTGCCCAGTGACCTCGGGGTTCTTGCAGGGAGCATCCCACAGGAATCTCCTGGCTGCGGGGTGAAGCAGCCACCTTCCCAAGACAAGGACTTTAAACACGGTCTGACAAACAAGAGCACATATGGGCCAAGGGCAAAAGAGAAAAAGTGCTAAGGAGGCGGTTTTCTAATTTAGTGGGTGTCAGATTCACAGGGGAGCTTATTAAATGCGGATCCCCAGGCTCCTTTCCTGGCGTTGATTCAGCAGGTCGGGGGGGTGGGGGGCAGGAATCAGCATGGTTAGCAAGTTCCTGAGAGTTCTTGGACTGGTGGTTTCCCAAAGACTGGGAGACAACAGTGCCCTATGTGGGCCCTGGAGGCAACCCCCCTGCCCAACGCATAAAGATGAGATGCAAGCATCATGGTTAGGTCAGGAGGGAATTTGACTTCCCCCAAAGAGTGAGAATGATTTGGTTGGAGAATGATAAAATTTATTTGGTCCAAAGGAAGGGAGGGAGGAAGAGAGGAAGGAAAATATAATTTACCAATACCTATATGTGCCAAGCTGTTACATGATGCTTTTCGTTTTGCTTTGTGCTTTTGTTTTTCAGAGACAGGGTCTTGCTATGTGGCCCAGGCTGGAGTGCAGTGGCACAATCAAATCATAGCGCACTGCAGCCTCGAATTCCTGGGCTCAAGCGATTCTCCTGCCTCAGCCTTCCGAGTAGCTGGGACTACAGGTGCATGCCACCATGCCTGGCTGTTACACGACATTTTTACCTGGCTGCTTTGACTTTTTGACATTCATATCCATTTTTGGAACCTAAAACGCTGTCAACATTCTATTTCTTATTACCAAGTAATGCTCATACAGACATACCCGCCACAGCCCGAGTCAAAACTTTGTGTCAGCTGGGATGTTGTGCACACCTTGGAGCATCTACCACATGCCAGGCATTGTGCTTTGCATGTACTGCTTTGTTTTTCACTGCACCGCAACACCGACAGGCAATACTATTCCCATTTTACTGATGAGGACAGCAAGGGTCAGAGAGGTTTAGTCACTTGCCTAAGGCCACCCGGCTACATCAACCCTGGCAACAGGAAGAGCTGGTGCTTTTGTTTTTTTACTGATCCAGGGAGAGGCCCTATTAGGGAGGTAGAGGGACTGAATCCAGATCCTGCTTTATAGCCCCCTCTCCAGGACTCCCTCCCTCCCACTCTGGAATGGAGAGGTGGATGGAGAGGCACCCTCTCTGCCACTCACTCAACCCCTCTGCCTTTGGGACCCTATGGACTCTCATACAGCCCACCCAGCTTGCACCCTGAGCTCTCTCCCAAGAAGCTGAGCACCTGGGGTCTCAGGCACTCCCTTCCAATTCAGTCCCTGGAGCACTTATGGCTCTGAGGGAGTCCCCGGAGGGGGGCTGAAAGAGGTGGGGGAGCTTAAGGAGCACCTTTCCTGGGCTTCCAGGAGCCTCTTACATCCCTATCCCCTTGGCAGAATGGGTGTATCACAACTGGAGGGTGGGGGTGTATGGGGACGGCAGAGCTGGCCAGACCTATGGTCCCAGATGTTTGTTCAGGCTGTGGGCTGAGCCTGATTTCAGATGTGTGGCAGCTTCATGGGAGTCCTGCCCAGACTCCCACATGGACACTCACTTGCTTACTTGCACACACGCGTGCCCTCATGCGACTCCCCTCCACACAGAGTTCCACTTGTGAAAACCCAGACAAAGCTCCAGACTAATGTCCCTGTGGACAGTGACATGAATGGTAAAAATCATCCTCAACCCCAGGTGCTCTGCACACGCTGACTCTAAACTTCACATCGATCCTGCAAGGATGCTTTCCCTGATGACCTCCCCCTACCCACCACACACCTTATAGATGAGAAAAATGGGCTTCAGAAAAGCCAAGTGACCGGTCTGAGGCCACACAGCGACCCCACGGCAGAGTGGGGACTGGGGTCTGTCTGTTGGGGAGCTCACCCGCATCCCTCTCACCAGGCTGCTTTTCCCGTCTGGCTCCCAGGACCCAGCAGGTGTTCAGCACGTCCACTGATGAATGTGTATGTCTGTGTTTTCTTTCACACACAGTGCAGGCTCCAGCTGCCCCTCTCTCCAAACCCACTCTGAGCTGTGATCTCCCTGCAGGCTCAGAAACTTGCAGACCACTTGGCTTCCTCTACAGCCAGAACTAGCGCCAGCCTGAATTCTCTGGGCTCCAGCCTCAGTTTCCCCTGAGTTTCTTAGGCCTGCACACCTGAGCAAGTCCCTCCCTGCTTTGAGCAGACCCACAGCACTGGTTGGGCTCCTGCTCCAGGCCTGACCCCCACTGTGGAAGCCTCTGGGGTCCAGCCACCTCCATTCTCCTCTCTTCTTCCCAAACGTCAGGCCTGGCACTGCCCCCGCTCAGCCCCCGCTCCCTACGGCCCCCTGGGAGCCCAGGCTTTGCTTGAGGGCAGCAAGTGAAGCCGGCTGTAACAGAGAAACCTCAGGCTCCTCTTTAGAAGTCCCCGGAGGAGGCAGATCTGAGGAAAGGCTGCCAAGAAGGCGCCTGCAGCAATGTCAGCTTGAGAACAACCCGGCTGACAGGATGCTGAGCGGGGATTAAGGTCGGGATCAGGGAAGCCAGGGAAATGAAGGCAAGTTCCCTGCTCCCCTCGCTGCCACCGCTGCCGCTGCGGCTGGGTTTGTCTTCTTGTTTGTTCTGGCTCTGGGAAACTGGGCGGGGAGGGAAGTGGGCAGGGATGAGGTGGGGAGGTGGAGGGAAGGACAGGGGGGAGGTGGAGAAGGGCTGGTATGGGGGTGGAGGGAGGTGGAGGGAAGAGAAAGTCAGGCCTCCTCTGGGTCTTGGGGCTCAGCCAGGGATGGGGCAGAGGCACAGATGGCCCTTACCTGGGACCCTTCCCCTATACCTGTCCCTATAGCTGGTCTGCCAGCCAGTGGAGCAGCCAGTCGCCCCCCGACCCCCACCCCGGAACCCTCCCTCATGTCCTGAGTGTGGGTGGCAGGACCAGTGAGAACACAATTGACCTTGCCCTGAGAGGCTCCCAGCTCAATGAAGCAGATGCAGTCTACCTCCAGAGAGGAGGTGAGCCTGTTTCCACCTGTCTACACATGCAAGATCCCCTGTTCTGGGGAAAGACCATTACCCCAACCTGCCTCATCATTGACATGTCACAAGAGTCCAGCCAAATGGGCTACTGATTTCCAGGTCATCCAGTGCACCACAATTCTAGTGAAACCTCATATCCTTGCATTGCCATTGGTGTGGTAGTGAAGAACGTGGCTTCTGGTTTCAGACTGCAAGGGGTCACCTTTTGGCTCTGCCACTGATTTGCTATGTCACATTGGGCAAGCTGGCTAACTTTCTATGCCTCAGTGGGATAAAATCATAGCACCTCCTCATAAACTTGTCAGGAAGATAGAGGGAGTGGACACGTGCGAAGTGCTTGGCATAGGGCCTGCCATGGAGGACAGAAGACATACGCCATAGGGTAGCTTTATTTATTATTGAGGCAGAGTACAGGAATAAGTGTGGCAATTTGAAGTGACTTGGCTGAAAGCTTCCTGGGGGAAGGGGTGTGGAAATCTGATTTTGGAAGCTGAGACTGGGCAGGGGTAGAGAAGTGAATTAGGGGTATGGAACAGAGCTCCCATTTGCCAGGTGAGTGAGTTAAGGATGTATCTGGTGAGAGGCAGAGAGGATGCATTTTCTAGAAGCCCTCATCCCAGCCAGGCTTTCTCCAAAGTTCCCACTTTAACTAGGGGTAACTCTTCCAGCCCTGCCAAGGGCAAGCCAGGGGTGCCAAGACCATGAAAAGATGCAGTTTTTCATGGTGACCTTCCCAGAATGGAGTCTATTCTAGAAACCCTAGCCATTCAGGGAGACTCCACTTGTACCTGGGTTTTCTGGACCTGGCAAGGGAAGAAACAGGCACAGAGAGTACAAACCTGGAGGCAGATCATCCCTGGGGTTGGGTTCCAGCTCTGTGACTGATCATCTATGTGCCTTGGAGCCAGTCAATTAGCCTCTCTGGACCTTAGCTGCAGGGTCTGTGAAATGAGATAGTAATATGTACCTTAGAGTTGTAATGATGAGCAGTTACTCCATAACTATCCACTGTTGATATTCAGCTTAGCAAAGAGTTGCTGAGGTCCAGCCAGCTACTGGGGCCCCAGTGAATGAGAGACCCTTTCCTAGCCCTTGATAGCTGGTGGCCTGCATGTGAGTCCAAATATGATGCTGAAGGCAGTGGTGCTACCTTAAAGGTCCACCTGGGAACCAAAGAGAAGGAGTTCCTGGCATCCACATCCCAGGCTGCAGGGCCTGCTCCCCTGGCCCACCCTGCAGCCCTGCTTGGGTGTCCCTATCTCTGGACACCCTGGCCAGCAGGCTGGGGATGGGGAGAGTGTTGTAGGGGGAGGGGAATGAGAACCTAGGATGGGGTTCTGGTAATTGGGGAAAAGCTTTGCTGGGTCTGATTTGTGGGGGAAAGCCTTAGGCAAGGAGGCCCAGTCCCTGCAGAAGTGTGTGAGGAGCACCTTATTGGGGATTTTATCTCTAGGGGCCTGGTTCTAGAATTTTAAAAACACCTTTTCTTTTCTTTTTGTTCTGTTAATAGAAGTTATGTATATTTATTGTTTAAAAAAATGGAAAATACAGGCCAGGCATGGTGGCTCATGCCTGTAATCCCAGCACTTTGGGAGGCTGAGGCGCGTGGATCATGAGGTCAGGAGTTCAAAACTAGCCTGGCCAAGATGGTGAAACCCCGTCTTTGCTAAAGCTATAAAAATTAGCCAGGCGTGGTGGCAGGTGCCTGTAATCCCAGCTACTCGGGAGGCTGAGCAGGAGAATCGCTTGAACCTGGGTGGCAGAGGTTGCAGTGAGCCAAGATCCAGCCACTGCACTCCAGCCTAGGTGACAGAGTGAGACTCCATCTCAAAAAAAAAAAAAAAAAAAAAAAAGGAAAATACAGAAAAGCATAAAAAAGGAAATAAAGATCAGCCATAATCTTCCCACCCAGAGATAACCATGGTACGACTTTAGTGTATCTCCTAGTTTTTTTTAAGCTGACTCTCAAACACACATTTACACACACTCACATGGACACACACATTCACACACACATATTCACAAATATACACTCACACGCACTCACACATTTACATACACATATATGTAACTTTTTGTTACATGTTTAATGTACACATGTCTTTAAAAGTTCAGAAACAAGATCATGGAATCATGAATACATCACCATTTTGTCAACTAATATTTTTATTAAATAAAATATTGATAACTTTTTCCCCAGGTCATGACATATTCTTCTCCAACATAATTTAAAATGACTGTATAGTGCTCTGTCTTTGGGCATAAGGCATATCATTTTATCAGTCATCACCTGTTGGTCATCCATCCATTTCTAGATTTTTGCCATTATAAATAATACTGAGGTGAACATCCTTGCAGAGAAATCTTCGAGGGGGTCGCTAACTTTCCAATTGTCTATTCATGTCTGTCTACTCCCGGCTTCGATTTTGAGGCTTTCTAGGCAGGATCTGTGTCTGGTTTGTTGTGACCCCAAAGTCTAGCTTGGCTGCTGGTTCACTGTAGGCATCCAATAATGGTTGAATTGAGTTGCTTTTCTAGAGCATGTGCCCTCGGCTGAGGAATTGGGAAGGTGGATAGATGAGGAAAGAGCCCACCTTTCCTGCTAGAACACTCAGGGGTCCCCAGTGCCTCTATGCTGGACCTGCTAACTTCTCCTAGGAAAACACCTTCCCAAGCACAGAGCCACTGGCCATCCCAGAGGCTCACTGAATGGCCCTCCTGTATCTCTTATCTTCCTTGCCTGGACCCTCCCCCCTCATCCCCTTCCCCCCTCCGCCCCCGATCTCTGACTCTTGAGTTCTTTTGTTGTGTTTTCAAGCTGAAGGCCCAGCAGACAGAGCCCTCTCACCTGCCCGTGTCTGCTGGACAATGTTGCGGCAGCTCCCAGCTCCCCATACAGTGGCCTCCTCCGTGGCCCCCACTTTTGACAGCACAGCCTGCTTGGCTCCTGAGAAGTGCCCACGTGGCACACCTCACCCCTGCCCCTGCTCTGCCTGCCACCTCTCATGGCACCAAAGGAGCCTTTGTCCCTTCCTCATGGGGGCTCTCAGCAGATGCTGACGTTCTGACTGAAACAGATTTGTAGAATCATGGATTTTGAAGTGGGGACCCAGGTCCCAGTGTTTCAGTGGGTCTTTAAAAAAGAGAGAAAGGGAGAGAGAGATGGAGAGAGAGAGAGAGAAAGTGAGCAACAGCACGAGAGGTAAGAGGCAAAAGCTGAGAGGTAAAACTTACTAACTTAATTTCCCCTCACCAGCATCCACACTCCCAACCTCCTGGAATTTCTTCTCTTCTTTTCAATTTTTAATCTCTTCTTTTCCTCTCTTTTCTCTCCCTCTTCCCCCTTTTCACTCCTTTCCTTCTCCTCTTTCTCTGTCTCTCTCTCAGTTCTGACATGAACACCCCCCCCACCCTCCTGTCCCCCTCCCCTCCTCTCCTCCTCTCCCCTCCTCTCCCCTTCTTTTTTAAGTTTTTTTTTTTTTCTTTTAACTTTCAAGGTCATTCCGGACTCCTGACGCCGCCAGTCCCGCGGTGAGACGTGAGCGCCATTGGCGTCCGTGGCCTCTGTTTCCGTGGCAACCTAGTAACCATTAATTTTCAATTAAAGGAGACAAAAAGCTCGATGACAGCTCCAGGTCTGCTGAAGATGTCAAGAATCTGTATTAATATACAGCAAGAGAGCATAATTGTGTGTCCATCTTCCAGAGCAGCGAAAAATGGAGGGATAATTACCAGCTCGAAAGCCACTCTGTAATTTAGTTCTTACTGTCACCACAGCCCTACACATTAGCATAAATTAAAAGCAGAGTTTATTGTTATCAAAGTGCA

**miR-423-5p (5’-3’)**

UGAGGGGCAGAGAGCGAGACUUU

**NACC1** **(5’-3’)**

GGGTGTTAATCACAGTGGATCCCCTGGGAGAGGACACGTCCCTAGTCCCATTTCACAGATGAGGAAGTTGTGGCTCAGAAAAGGGGCGGTAAGCGGAGAAACCCAACACTTATTGGTCCTCTTCTGTGTTCTGGGTGCTATATACACATTATTTCAGAGCACCCTGCAGGAATGACTCAGGCTCTGGAGTTGGATTTACCGGGTTTGAATCTCAGTCCCATCACATGCTCTGGGCAGGTTACTCCCGCGGCTTCACTTTTCTTACCTATTAAATGAGAATCGTGGTTTCTCAGATAGAGCAAAGATTAAATGACAGATTCCTGGAAAGTGTGCAAAGAACCACTCTAACATTGTAAGCGCTCGGTAAAGTTCTAACCAGTGTATGAAGGCAGGACAGCGAAGTGGTCCCGGCTACCTGGGTTCCAGTCTCAGCTCGCCCTGCGACTTGTTAACTCTGAACTTAAGTTACCGCTTTTGCAAAATGGGCAGAACTTTTTTTTACCATTTGTTACTGGGTTGTAGTGAGAAGTAAACGAGTTAGTGCAGGTAAAATGCTAACAGAACCTAGCATCCGGTAAGCATCATAAAAGTCTTAGGTCTTATTGTCGCTTTTGAGCCTGTGTTTAAATCCTGGCACCTACCATCACTTCCTTGCTGTGTGATGATCTTGACCAGCTTTCTTCATTTCTCTGTGCCTCCTTTTCTTTCTTTCTTTCTTTTTTTTTTTTTTTTGAGACGGAGTCTCGCCCAGGCTGGAGTGCAGTGGCATGATCTCGGCTCACTGCAACCACCGCCTCCCGGGTGCAAGCGATTCTTCTGCCTCAGTCTCCCGAGTAGCTGGGATTACAAGCGCATGCCATCACGCCCGGCTAATTTTTGTATTTTTAGTAGAGACAGAGTTTCACCATATTGGCCAGGCTGGTTTCGAACTCCTGACCTCAGGCGATCCACCCACCTCGGTCTCCCAAAGTGCTGGGATTACAGGCGTGAGACACCGCGCCCAGCTGCCTTTATTTTCTATAGTGTGTCTGTAGCAGTTCTCACTAAAAAATGTTACTGTGTAGTTTAAACGAGCTAATTCCCATAAAGAATGTAACGCAGTGCCTGCCACAATAAACAGCAGTCATTATTGTTTTTGTTTTACAGGAAACTAAAGTTCGGAAAGGTAGAGCGACTTGCCCAAGGTCGCGGGACTAGAAGGTGGCGGGGCCAGGATTCGAACCATCGAGCTGAGCCGACGTACTGGCGAGGATCCCCCAGGGGCATAGAGAGGAGACGGAGCCGGTTAGAAGGGGCGCGCTCGGAGCTCGGCTGGCCTCGCCTGGAGGGCGGGGCCCGAGGGCGGTGCGCGGGCGGGGCCGGGGCCGCTCCTCCCTGGCCTGGCTGCGGCGGCAGCGCGGCTTGCGCTCGCTCGGCGCTCGGCTGGGGCGGCCTGGCCCACAATGAATGGCCGCCGCGGCTGCCGCTGCTGCTGAGGCGGAGGCCGCGGAGGCCGCGGAGGCGGAGGCCGAGGCCCCGGCGCAGCGGGGCGCGCCCCGGGCCCAGGCCCGGCCCCAGCCGCCGCTGCGGAGCCCGCCGGGACCCCCCGGAGCGCGGCCACGGCGCAGGTGAGGCGGCCCGCCTCAGGCCCGGTTCCGGCCTCCTCCAGCCGGGGCGGCCCGAGGGCTGAGTGGGGAGGGCCTGGGTGCTTTCTCGGACCGAGGAGGAGCCGGGCCCGCAGCCTGCACTGTGCCCGGGCAGGCGAGTGTGTGGAGGCCCAGCTACCGCGGGCGGCCTTGAGCGTCCAGCCCGAGCTTCAGGGCTTGGGGGCGGCAGGATCGTACCTGGACCGCTCCCCCGTCTCCCAGCGAGGGAGGGGGCCAGGTCGGGCCGGGGCAGGTACCGGGGATCCGGGGAAGTTGGAGGGTCCGAGGCGGAAAAGTGAGGAGGTTCTCGTGGATGCCGGAGGGGGAGGGGGAGGGGGAGGAGGAGTCGGGCTCCGGCCAGGCCGAGTGGGGGAGGAGAGGAGAGCGGCGCGGCCGGAGGGGGGCGGGGAGGGGCCGGGCCTGCAGGTACCGCCTGGGCGCTGGAGGAGGTGGGTGGGTCAAGTTACAGGCCTGGGGGCGGCTGAGGAGAAGCCCAGGAAGTGGGGCACGGCGGGTGTGGGCGGAGTTACTTCGTGTGGGGGCGGATAGGAGATCCCAGAGATGTTTTCCGGGGGTCACATGAGGTCAGGACTCAGATGTGGGGAGGGGGAGGCTAGATGTGGGGGCGATCAGGTAAGGTGAGGGACTGCTGTAGGAGTTTTGGGAGTCGAGAGAGCAGAGCCTTTTGGGCTAGGCTAGGGGGGATGTGGGGGCAGGTCAGGAAAAGCTGGTGTGACCAGAAGGTATATACTAAGATGCGCCCCCCACCGCCCCCAACTCAAGTGTTAAGGGGTTCCGTCTTTCTCACACCTCATCCCTGAAGCCTCTAGAGTCCTGACCCTACCCCTTGGGCAAGCCTCTGCCAACTGTTCAAAGGCACTGGCCAGTGGGTGTTTGTGTGGGCAGCTGGAGACAGTCTGGAACACCTAGCTTAGGCATCTTGTTTATCACAAGGTGGCCCTTGCCCACCCCCACCTTATATATCTACAGGGAGGCAGCCCAGCAGACGTTACCTGGGTATTTTAGGATCCTGCATTCTTTCTCTACCTTTTGTGGCCAGAAGCTGGTGGTTCAAGCCTAGTGGATGGGACCTCACAGCAACCATCGTATATATTAGTACCATGGCCTCCCAGGCACCAACTCCTTGAGCCTGAGTTCTGTGTGCCATCTCCCTGGGGGTGAAGAATTAGAGGGAATGAGCATGTAAAACTCTGTTGATGGTGGTTTGGAGGCTCCCGACGGGGTGAAGGCAGGGCACAGAGAAGAGTTAAGGGGATCAAATCAGGCACTCACACCCTGGCCCAGGGATCTACCTCTGGGACACTCGTCCTGGGCCAGGCCCTGTGCCGAGCTCGCTGGTCGGATGAGCTCATTGATGGCTCACCACCACCAGTTCAACTTAGGCCTTCTCTGGGGTCCAGACGAGGAAGCTGAGGCTCGGGGTGGTGTGGCTTGTCTCAGATCACATGAGCAGACAGTGGCTGAACTGGGATTGTCTGACCCTCAGCCCAAACCCATAGTCACTCAAGTGGCCTTCCAGGAGGGTGACTGAAGGAGCTCCTCGTTCTGGATTCCCTGGCGGGCGTTCTCCTCCGTCCAGGCCTTCCCTTCTGCTCACCCCGGGGGATGGGTCAATCCCCTTTGGGACAGACTGAGTTTCCATGGTGTCCCCGGGGAATGGAACAGTGGAGGTATCTTTCTCGGAGGCCTGGTCACCCCTTCTTCCCCATTTCCCTCCTGGGAGCCAAGAGGTCAGGAGTCATGGAACTGTGGGCTGACCGTGGGAAGCTGAGGAGGCCTGGCACCACCAGGGCTGGACTTGACTTTGCTTGCCTAGACTGTTTTTGCCCCACTTTGGCCCCCAGTCGGATGGCAGCAGCTTTTGTTCCTTTCTGGGCTAATTGCAAGGCTGTTACCAGGGTGTCCTTGGGACTTTGATTTGGGATCTTCAGCACCTGAGGCAGGAGCCACAAACTGCTGGGTCAAGTGGTGTCAGCCAGTTCTGGGTTTAATGATGGGTTGTCACCCTAATTCTCCCCAGTCTCCCTATAGTTCCCAGGACACCCTCCAACCTGGGCCCTGCCCTTCTTGCCTGTCTGGCTAGCCACTTGGTTCTAGGAGGAGTAGGAACTGGGCCAGCAGTCCCGCAAAGAGACTGAACCATCCTTCCTGGCTGGTTGGGCCAGGATTGCTGCAGGTCTGCTTCAGCACAGACTGGGTCCTCCTGGACCTAAGCTGGGTTGGATACCAGGGATAGGGGATTGGTGGTACCATCCAGTCACTTAGCTGAGACTGTTGGGAGTTGGCCCTGGTGAGTGGGGGACAGGCAGTGCCTCAGGCAGCTCCGGTTTGTGCCTAGAGGTAGAGGAGAAGGCACCAGTAATAACTGCTTTTCGGTGAGAACTTAGCATGCACAGACTCTGTGGGGGCAAGTGCAACAAGTAGGCAGCCCGTTGAGACAGTTTTATTTCACTGCTGGGTGCTGACTCCCCGCTGGGGGCTGGGGAAGCATCCCGGAGTAACAGATCCAGAGCAGCTAGCTCTCAAGCGACCAAACTTTCACAAGCAAAGGATCAAGATAATTTCACATACTTCTGTGTTTACAAAGATGGTGTAGCAAAGTGAAGTCATTATGAGCCATGAGGGGATGGGGAGGTTTCGATGGGTGGCCGGAAGTGTTCCCGGAGAGCTAAGATCTGAATAATAATGTTAGCTAAGGGGTGGGTGGTGCTGGGGCGCAGGGAGGGGTCAGTGTTCTAGGCTCAGGGGACAGCACATTCACAGCTTTTCTTGAGTATGTACCAGTCTTCTTCTGAGTGCTCTAAACATTTTGTCTTCATGAGGGAAGGAGTGGGGGGATGACCCTACTGTGGAGACAGAGACTCTAGTACCATTCACAAGAGAGTGAAGCCCTGTCTCTGGGTCTCCTGGATCTGCCCATCACCCCCAGATGGAAGTTCAGAACCCCTTGGCCTGCATCTAGGGTATTTAAAAAATCTGGCCTCCTGGCCGGGCAGCAGGGAGCCCCAGCAGGTTCTTGAGCTGGGCTTTGAGGAGATGTGAGTTTTGGTCAGCCTTACAGGGTCTATTGAAGGAGTCTGCCTGGAGGCCCGGGGGTTTGGAGAGAGTTTGGGGTTGGCCCAACATTGTCTGACCCTAGATTTCATCAGTGTCATTGCCAGAGCCTGTGTGGAGAGAGGGATCCCATCAGGCTTGGTGGGGCATATTGCTTTTGGGATGGTGGTGAGCCACCGGGGCTGGAAAACATGAGTCAGTGTTCATGCCACTCTGCGTCCTCAGATCCTCCGTGCCCCTCGTTTCTCCTCCTAGGTTGCTCTCAGCACTTGTACACACTCGAACTCATTTAAGCCCGTGGCTAGCAGGTCCTGCCTGTTATCATCATTTGACACACAAGGCAACTGAGGCACCCTAGTCCTTCCTTCACACAAGCTGCAAAGTGGTCTGGCTACCACCCCTGTGCCGCCTTCCAGCCACTTTCATGTTCCTCGTGTCTGCATTTGCTCCTCAGTCCTCTCAGCCGCTACTGCCTTCCCCTAGAGAACCCTCTGGGCCGCATGAGGTGGGGCCGGGAGGTGGGACTGGGCCTGCTTGCACATTAGGTGGGGTGAGGACCAGCCGAGCCGGCAGAGGTGACGTGTCTTAGGTTGTGATCTGTGTGTGGTTGTGGTCATGTCCCTCCAGCCTTCTCATGTGCTGCCTCCTTGGCGAAGGTCTCAGTTCTTAGAGACCTGTGCGTCTTCTCTGAGGGCTTTGACTGTGTCCAGTTCGTGGTTTCTAAATCGAGCAAAGGGAGAGGGTAGGTAAGGGTGAGCAGACACGGTCTAGTAGAGAGTGAAATTTCACACTTAGCACCACCCCTGTCTCAGCCATGAGACTCTGGGCAAAGGTCTTTACCTCTGAGCCTCAGTGTCCTCCTCTGATAAGGAGTGACAAGTGGGCACAACTATACTGCTGTGATTCGCAACTGGGTACAGTCCTACCTCCGGGGACACTTGGGGAATGTCTGGGGACATTTTTGGTTGCCCCTGCCGGGGGGGGGGGGGTGTGCTGCTGGCATCTAGTGGGTTTATAGGCCAGGGATGCGGTTCAACATCAGACAGTGTGTAGGGCAGCCCCCACCACAAAGAATGATTGACGCAAATGCAGTGGCTGGGTGGGGAATCTCCACTGTCTGGTTATCATGAGGTTTGCGGGAGAGGTTGGCGTCTGGGGTGTGAGTGGACAGTGCTGTGAAGGCTCTGGGGAAAACCCCAGCGATCACTGCCTGTTAATCCCATGAAATGTGCGAGGTCTGGGAGATGGTGAGAGGCATAAGATGTCTTCTACTCCCAGGGAGTTTGCGGCCCAGCACGGGGCCAGGGGATACGACTAACAGCAGCTGGCATTCATGAGCACCGACTACGGGGCGAGCGCCGATCGGCCTGTGTTCTTTGGTTGTTTAATCCTTGAAATAGCCTTGTGAGGTGGCTGCTGCTGTCAGCCCATTTTCTAGATGGGAGACTGATATGGGGGGTGAGCAGTATTGAGGGTAATTCTGAGTGACAAGCTCTATAAGGGATTTGAGCCTAGTACAATAGTAGGATTCAAAATCTTAGGTCCTGCGTGGTTCCCTAGCACAGCCCAGCAGGCCCCTCTCCCAGGATGATCCCCATCCCTCCACCCTCCTTTGTGTTAGCGGCACCAGGGTCACCTCAGGGCCTTTGCACTTACTGTTGTTCCCTCCACCCTTGGTTGCCGCCACTGCTGCCCTGTCTTTTGGGTATGATCTGATTACACGCAGGGAGTGGGATGTAACTCCGAAGGGTGGGCTTATGGTTGGAGTTGGTGGGGAGGGAGCGGGCAGGCTGCGAGCTCCATGAGGACGGAGGCAGAGCTTCTGCCAGGCTCTCCAAGGAGCTGGAGCTGCTGCCTGCAAAGGGAGGCCAGATTGCCGAGGGCTGCGAGGAGGAGCGAGTGGGAGGAGGGAGAGGCAGCACGCGGAGACTGCTCATTCCCGGAGATGGTGGTTGCAGGGAGAAGGGGCTAGAGAATAGCTGCAGTTGGGAGGATTGGGAAAGGAAGAATGAAAAGAATGAAGAAGGCCTTCCTTGGGGATTAGAGAGAGGGCACCCCAGACCCTGAGGTGGAGGTCCTCAGACAGGCAGAAGACCCTGTTTCTGGAGAGGACACATCCAGTAGCTCAGGTAAAGAGCCCCAGCTCTGGGCTCGGCCGGTCCGGGGTTCCCGTCTGGTCTCTGCCTTGCTTCTCTGAACACCAGTTTTCCCACCTGTGAAATGGGTCTAGTCACAGCTGCCTCACATTTTTGCCTGATTCGGGAGCCAGACGGCCTGGGTATAAACCTGTGCCTGCCATTCACCAGCTACATGTTCCCCACCTCTGGCTTCACCTCTCTGGGCCTCATTCCCATCCATGAAATGGCAGGCAGGCTCATTTCTTCCTCAGAAACTGTAAAGAAGAGTAAGTTAAATGAGCCTGTCTGAGCCCTGGCCAGGTATGGGCCCAGTCAGTGAGGCTTGAGTAGGGGACACTGTTACTAATCCTATTCCACCATGGATTTAACAATAGTCATTCAGGGAAAGAGATAACGAATTAAATCTGTGGGTGTATAAGAAAAAAAATACAGGCCAGGTGCAGTGGCTCACGCCTGTAATCCCAGCTCTTTGGGAGGCTGAGGTGGGCGGATCACTTGAGGCCAGGAGTTTTGAGACCAGCCTGGCCAACATGGAGAAACCCAGTCTCTACTAAAAATACAAAAATTAGCCAGGCATGGTGGTGTGTGCCTGTAATCCTAGCTACTCAGGAGGCGGAGGCACGAGAATTGCTTGAACCCAGGAGGCGGAGGTTGCAGTTAGCCGAAATCACGCCACTGTACTCCAGCCTGGGCGACAGAGGAAGACTGTCTCAACAAAAAAGAAAAAAAAATACTGATGCAAGATGAGGTCTTGCTGTATTGCCCAGGCTGGTCTCAAACTCCTGGCTTCAAGCGTCTTCCTGCCTCAGCCTCCCGAAGTTCTGGGATTACAGGTAGGAGTCACCGCCCCCCACTGAACAGTTTGTTCTTCACTAACTGTTTTCCTGGACTGCCTTCACATTCAGCCCCTACAGAGGGGTCTTTCTGGTTCTGGGTCAGGCAGGCAACCTGAGTAGCTGCCAGGATCCAGCCACATCCTGCTGAGCTGCATGAAGGACTGTGGGGCTCTCTGGGGCCTTTGTTCCTTGTTACACTTGGAGTTGCTGGTCTCCCTTAACTGGCTTGTAGGTCTTAGCCCATCTGGGCTGCTGTAATAAAATACCTTAGACTGGAAGCCAGGCACATTGCTCATGCCTCTAACCTCAGCATTTAGGAGGCTGAGATGGGAGGATTGCTTGAGCCCAGGAGTTCGAGACCAGCCTGGGCAACATAGTGAGACCTCATCTTTACAAAAAAATTTAATTTTAATTTGTTTTTCACCCGTGACACAGCCCTCAGGAGGTCCTGAGAACATATGTCTCTCGTCTTTGCAAAAAATTGTTTTAATTAGCCGGGCGTGGTGGCACACATCCCTAGTCCCAGCTACTCAGGAGGCTGAGGTGAGAGGATAACTTGAGCCCAGGAGTTTGACGCTATAGTGATCCAAGATTGCACCACTACGCTTCAGCCTGGGTGACAGAGCAAGACCCTGTCTCAAAAAAACAAAACAGACTGGGCCATTGATAAACAACAGAGATGAGCTGCTCATAGTTGTAGAGGGTGGGAAGTCCCATATCACGGTGCTAGCAGATTCCGTGTCTAGTGAGGGCCCATCTCTCATAGATAGCACCTTTTAGGTATCTGCACAGGGTGGCAAGGACAAACAAGCTCCCCCTCTTTTATACGGGCACAGTTCGTATTCATGAGGACAGAGCCCTCCTGACTGAATCAACTCCCGAAGGCCCCACTTTTTTTTTTTTTTTTTTTTTTGGTGAGACGGAGATTTACTCTTGTTGCCCAGGCTGGAGTGCAGTGGTGCGATCTTGACTCACCGCATCCTCCGCCTCCCAGGTTCAAGCGATTCTCCTGCCTCAGCCTCCCAAGTAGCTGGGATTACAGGCATGTGTCAGCATGCCAGGCTAATTTTGTATTTTTAGTAGAGACGGGGTTTCACTGTGTTGCCCAGGCTGATCTCGAACTCCTGATCTCAGGTAATCCGCCTGTCTCTGCCTCCCAAAGTGCTGGGATTACAGGCGTGAGCCACTGCACCCAGCCCTTTTTAAATTTTTGATCAACAACTTCTTTTTTATTTTGAGATGGAGTCTTGCTCTGTTGCCCAGCCTAGAGTGCAGTGGCGTGATCTTGGCTCGCTGCAACTTCCACCTCCTAGGTTCAAGCAATTCTGCCTCAGCCTACCTAATAGCTGGGATTACAGGCGTGCACCACTGCGCCCAGCTAATTTTTGTATTTTTAGTAGAGATGGGGTTTCACCATATTGGCCAGGCTAGTCTCCAACTCCTGACCTCAGGTGATCCGCCCACCTCGGCCTCCCAAAGTGCTGAGATTACAGGCGTGAACCACCATACCTGGCTGAAAGGCCTAACTTTTTTTTTTTTTTTAGACGGAGTCTTGCTCTGTCACCAGGCTGGAGTGCATTGGCCCGATCTCGGCTCACCGCAACCTTCGCCTCCTGAGTTCAAGCGATTCTCCTGCCTCAGCCTCCCGAGTAGCTGGGATTACAGGCACGTACCACTGTGCCTGGCTAATTTTTTGTATTTTTAGTAGAGATGGGGTTTCACCATGTTAGCCAGGATGGTCTCTATCTCCTGACCTCGTGACCTCGTGATCCGCCCTCCTCAGCCTCCCAAAGTGCTGGGGTTACAGGCGTGAGTCACCGTGCCCAGCCAGCCCCACTTCTTAATACCACTACATTGGAGATAGGCTTTAGCATGTGAATTTTGGGAAGAGGCAAACATTCAGACCATAGCATCTGTCGACGGCCATGTCACAGCTTCCCTGGAAAGACATCAGGAAAGGACTGTGCCTTTGACAGTCTCTCACAGGCCCATGCTGGCTGGGGAATTCCTCAGCTCTCTTCCCTAAGAGCTGACAGTTACTACTCCCAGGAATCATCCTGCCAGCCCCCTTCCTGCAGATGGCAGGATCCACCGGCAGTGTGAGCAATTCGTCAGGTAACACTGCGGTCTCAATAGGCCCCTGACTGCTGGCTACCCACCTTGGCAGCTGTCAAAGGAGGTCGTCTGTCTGCCCAGCCACAGATAATGCTTCTGAGCCCTATTCCTGTTTGAGCCACGTTAATGTGGAACAAAACAGGGAGTTAGGTTAGGAGGAATGGAAGTAATTCTTTGTTCATTTTTTCCACGAAGGACTGTGAGCACCCACTGTGTACCTGGCCAGGTCCTAGGGGTAGAGGACACAACAGGGATCTACACAGGCAAACTCCCTGCCCCAGGGAAACTGACATGAAGCCCCAAAAAATGCCCAGTTTGGCAGGGGTGGTGGCTCGCACCTGTAGTCCGAGCACTTTGGGAGGCCAAGGCAGGAGGACCACTTGAGCCCAGGAGTTTGAGACCAGCCTGGGCAACATTAGGGAGACCTCGTCTCTATTACTAAAAAATGCACAAATAAATAATTTTTTTTTAGAAAAAGAAAAATGGCCAGCTTGCTAAATAAATCATAGGAACTCCCAAGAAGGATAAGTCAAGCAGGGAGAATCAAGCCAGGGAGTTAGTGTCGGGAAGGGCTTTGTTGGTCAGACGCAGTGGCTCATGCCTATAATCGCAGGGTTTTGTGAGGCTGTGGCAGGAGGAACACTCAGCTGGGAGTTGGAGACCAGCCCAGGCAATATAGTGAGATCACGTCTCTACCAAAAAAAAAAAAAAAAAAAAGCATACATATACATATACTTTTTTTTTTTTTTTTTTTTTTTTTTTTTCGGTTAACTGGATGGGCCGGGTGCCGTGGCTCACGCCTGTAATCCCAGCACTTTGGGAGGTGGGCGGATCACTTGAGGTCAGGAGTTCAAGACCAGCCTGGCCAACATGGTGAAACCCCATCTCTACTAAAAATAGAAAAATTAGCCAGGCATGGTGGTGGTTACCTTTAATCCCAGCTACTCAGGAGGCTGAGACAGGAGAATCGCTTGAACGATTCTTGCAGTGAGCCGAGATCCCACCACTGCACTCCAGCCTGGGCGACAGAGCGAGACTCTGCCTCAAAAAAAAAAAGAAAAAAAAAAATTAGCTGGATGTAGCTCCTGTGGTCCCAGCTATTTGGGAAGCTGAGGTGGGAGGATTGCTTGAGCCCAGGAGTTTGAGGCTGAAGTGAGCTATGATGGTGCCACTGTACTCCAGCCTGGGTGACAGAGCAAGGCCCTGTCTCCAAAAGCAAAAGAGTAGAGAAAGGGCTTTATGTGACCAGGGAGAGTATGAAGAGCTATGGCTGTCCCATTCCCATTCTAGTAGGTCCGAGAGCAAGCAGGGCCAGACTGCCAGGGGCCCCAATAAAGCATTGTAGGGGCCCAGGCAGTGTCACTTGGGGCCAAGGTAGTACAAAGTGGTTTTGAAGGAGTTGAAGATGAATCCAAGGATCTGAATGAGGAGGGCCAGAGCTGGTGAAGCTACGGCATTCACAGGTTTTGGGAAGGACTGGAGCCAGGTCCTGAAATTGTTGAGTGGAGGTGTCTGGGGTGTGTGCGAGTCTGGCATTCACAGGGAAGTCCGGGCCAAAGACTCCAGTGGGGGAGGCATCAGTGAATAGATGGTGCTTCAGGCCTGTCTTAGTCAGCTCAAGCTGCCACACAACAAAATATCACAGACTGGGTGGCTTAAACAACAGACATTTATATTTCAGAGTGCTGGGGGCTGGCAGTCTAGATGAGAGTGCCAGCGTGGTCAGGTTCTGGTGAGGGCTCTCTTCATGGCTTGCAGACAGCTGCCTTCTTAATACCACCGTTATGACCTCAATTAACCTTAATTAGCTCCCAAAGGTTTTATCTCCAGATACAGGCACATTGGGGCTCAGAGTTTCAATCTGTGAATTTGGAGGGACATACTTTGTCCATAGCAGAGCCCTGAGACTGAGCCCAGTCCCCTGGAGGTGAAAACCCTTTCTGGAAGGGCCACACTCCCCATCCACTACTGCCCTTCCTAGATCTCAGCTCCAGTGTCACCCCTTCTGGGCAAGGTCAGGCACCCCAGTGTGTAGCACATTGATCTGTGTGACCAAGGATGAATGGCCCTGTCTCCTGCTGGACTGTGAGCTCCAGAGGCAGGGCTACTTTGTCACCATTGTGGCCTGCCCAGGCAGGGACCTGGCAAATATTTATTGAGTAGTTGTTGGGAAGTCATGGAAACCGTGTGCTGTGCCCTTGGGTCTCACCAGAAGATCTGCTCAGGCCCAGGGCCAGGACTAGGCACTGTCCTGGCCAGGAAGGTCCCAGCTGCCCTGTGGCCTCCTGCCCTTGTCCCCCACACCCTCAGGGGTGCTGATATGCTGAATTTTAAGGAGAGATGATTCTCATATGGTTTTCTGCCCTTGGGGAGTTTATAGTCAAGTGGGACTTAGTTTCCTCAGAGGTAAGCTTAAGGGCACTGTGGCCGGGGTGGGGCAGGAGAGGGACATCTTCTCTAGCCTGGAGAAGTGAGTAGCTGAGGGTGGAGGAAGAGGGTATTCCAGGCAGGGTGCACAGCTTGGGCAAAGGCCTCGCTGCAGTAGAGGAGGACCAGGCAGTGTGTTTGAGCCTGACTTAGGTGGGGATTTGGTGGGGACTAGAACCATTAGGTGGTTTAGAGACCTCAATCCTCGACTCCATCCCACCCCTGCTTCAGCCAGCAGGTGACCCTGCTAGGGACCTGCCAGCGAGAGACCCGTTTCCTCTGAGTCCAGTCATGCTAGCTCCCTTCCTGGGGCTAGCGTGGGTGAGGGCTTCTTGCAGGGGCCCACATAGTTTTTCTCCTGAGTCCATTGGGCAGGATTGGGCCGGATTGGGCCAATCACAGAGGGATGGAGGGGTTCCCAGAGATGGGTCTGCTCTGGCCCACACTGCAGCTCACCCCTGTCCACTCTGTGCGGGGGCCACTGGGGAACTAGACGAACAGCATTCAGGTCACTGTGGCTCACAGACCCGTCGCCCCACTCCGCTCGTCCCCAGCAGCATGGAGGGAGGGAGAGCTCCCGTGCGCTAATTGGCTGAGCTATTTGGACCAATTGCTAATCAAAGGCTCCAAAGCCCTGGGTGCTGTGGTGCTGGGCTCTGAAGCTGAGGTTCACAAGGTATGGGGGACCTCAGTCTGAGCATCAGGTTGGCTCTCATTCTCAGGAGGGGACCTGGGCCACCTTCCCAACCCCCACATCTCTGTTGCCTCCTATTATCCCCGTAACCTTTCAGTCTCCATTGCCAAATGGGGGGGTTCAAGACCAGCCTGGACAACATAGTGAGACCCTGTCTTTAAAAAAAAAAAAATTACCTGTGGCCGGTGCTGGGTGCTTGGTAAGGTCACCTTGAAGCAGTCCTGTGCCCGTTCTGCACTGTTTTGTTTGTTTGTTTGGTTGGCTGGTTGGTTGGTTGGTTTTGGTGGGGTAGTGGGGTCGGGGGATGTGGTTTTTTGTTTTTGCTTTTTTTGTTTTAGAGACAGGGTCTCGCTCTATGACCCAGGCTGAAATATAGTGGCACAATCATCACTCACTGCAGCCTCTAACTTTTTGGCTCAAGTGATCCTCCCACCTGAGCCTCCCAAGTAGCTGGGACTACAGGTGCAAGCCACCAAGCCCCACTAATTTTAAAATTTTTTGTAGAGATAGGGGTCTCACTATGTTGCCCAGACTGGTCTTGAACCCCTGGCTTCAGGGGCTCCTCCTGCTTCTGCCTCTCAGAGTGCTGTGATTACAGGCGTGAGCCACCGTGCCCAGCCTCTCTGCACTGTTCTTTAACACATTTACAAATATTTACTGAGCATCTATTTCATTCTAAAGAACTAATCTTGAATAAAACCAGCCAGAATGTTTGCCTTCATTGAGCTGACAGTGCCCTTGGAGGGGTTAACTGTACATACACCTCTTTTTTCTTTTCTTTTTTTTTTTTTTTTTTGAGATGGAGTATCACTCTGTTGCCCAGGCTGGAGTGTAGTGACTCGATCTCAGCTCACTGCAACCTCCACCTCCCAAGTTCAAGTGATTCTCGTGCCTCAGCCTCCTGAGTAGCTGGAACTACAGATGTGCACCACCACACCCGGCTAATTTTTGTATTTTTAGTAGAGACGGGGTTTCACCATGTTGGCCAAGCTGGTCTTGACCTCCTGACCTCAGGTGATCTGCCCGCCTCAGCCTCCCAATGTGCTGGGATTACAGGCGTGAGCCACCGCGCCTGGCCTGTACATATAACTCTTAAAATGCTGCCCTGACCCTGGGGCTCACTTTCAAAGATGTCCACACTCTGTAGGCCAGGACCCCACCTGTCCCATACCCTTCCTGGCAGCCCTGTCCCTGCTACATCGCCCTTCAGCTACACCAGCTTTCTTTGGGTCCCTGGACCACACCTGATACCTTCCCGCCCAGAAGTGTTATCTCTGTCCTGGCAGTTGCTCAACCTTTTATGGTCCCTACCACCAAGGCCATCTGCTTGGTTGACATACCCACCCCAACTGTAATTAAATCATCCCCGGTGTCTCCTCACTAGACAGCTCCACATAAGCACAGCTGGTCCTTTTAGGGCTGTGTCCCCAGCACCTCCCACAAAGGAGGATGCAGTTCATCAGGATGGGTTGGATGAACAGCCCATTTTACAGATGAATAAACTGAGGCACAGGCAGGTGCAGGATTTTGGCAGCACTGCACACCTGTCCTGGCTTTGTCCTCCTCCCTGGGGAATGAGCTTGGAGCACCCCTCTAGGAACTGGGATGCCAGCCTCTTCCCTTGACCATCTTGGTCCTTGTCATGTGGGCCCCAGTACCAGGGGTGGCTTTAATTTGGCTGCCCAGGGAGGTGGTTCAGGGCTGCCAGGACCCCTGAAGTAGGCTCTCTGGGCTCTGTCCTTTGCCCCCCAACATGGTGCCTGTTTCTCCTGCCTCTTCATGCAGAGTGGCCAGCTCCTGGCCCATTCAGCTCTGAGTTTGAGCTCCTGGGCGGAGATTTTTGTTGCCTTGTAACTGGGATTCCTAGATCCAGCCTCCACCCTCTGATTGCCCTAAGGAGGTTTTGGGGTTTTGTTTTGTTTTGTTTGTGTTTTCGTAGGATGGTGGCAGTGATTTTGAGGAGGGGAAGAGGAAGGAAGATACAAGCAAGGGAAGTCACAGGCTGTGGTGCCTAGGCAGACCCTGGTTTGAATCTTGGCCTTACCACTTTTTGCATGTAAGTGGTAGGGGCAGCAGATAGCTCAGGACATATCTTGGGTTTTTTTGTTTGTTTTTGTTTTGAGACATAGTCTCACTCTGTTGCCCAGGCTAGAGTGCAATGGCACAATCTTGACTCACTGCAACCTCCACCCTCCCGGGTTCAAGTGATTCTCCTGCCTCAGCCTCCCGAGTAGCTGGGATTACAGGCACCGGCCACCACACCCAGCTAATTTTTGTATTTTTAATAAAGATGGAGTTTCACCGTGTTTGCCAGGCTGGTCTCGAACTCCCGACCTCAGGTGATCCACCTGACTCGGCCTCCCAAAGTGCTGGGATTGGCCAGGTGTGGTGGCTCATGCCTGTAATCCTACACTTTGGGAGGCCAAGGCAGGTGGATCATTTGAGGTCAGTAGTTCAAGACCAGCCTGGCCTATGTGGTGAAACCGCATCTCTACTACAAGTACAAAAATTAGCCGGACATGGTGGCACACGCCTGTAGGCCCAGCTACTCAGGAGGCTGAGAATCACTTGACCCTGGGAGGCGGAGGTTGCAGTGAGCCGAGATGGCACCACTGCACTCCAGCCTGGGTAACAGAGCAAGACTCTGTCTTAAAAAAAAAAAAAAAAAAAAAAGTGCTGGGATTACAGGCTTGAGTCATCCCGCCCTTCAGGACATATCTTTTGAATTTTGAACCTGGTGAATGAATTTTTAGATGACCTCAGGACAGTCCTCAATTTCCACATGACCTCAGTTTTTCCAGCTTTGCAAGGGGAGTATGAAACCAGCCCTGAAGTCTTGCTAAGAGGATTCGGGGAGCTCATAGAAGTTGTGAGTTGGTTCCTCCATTCGCCTGGGTGTTGTAAGGGGCAGGGCCTGTGTCTTGCTTGTGGCCTGCCAGGGTAGCAGCTCAGTAAGTGAGATGGTATCTCACTGTGGTTAGGATTCACATTTCCCTGATGACTAGTAATGTTTCCGTGTAATTTCTTGAATGATCATTCAGTAACAATAAGTGTAGGTTGAGTTTGTAGAATCAGGTTGACCTCCTCAGCCTCGATTTCCAAATGGAGAAAGCAGAGATTTGGAGCTCCTGGTCAGAGGAGCAGGTCGGTGCTGGAGCCAGGACTCAACCTTAGGTTGTACCTCTGAGCCTTTGAACACTTGCCGTTTCTATTCCTCACAGCAGCCCTTTCAGACGGAACGGGTGTTACCAGATTAGGAGGAGAGTGGCCACTGTATTCTCAGTGCCCAGCCCAGGGAGGCAGATCTGAGTGGAGTTGCTAATGAAACATGGAAGGTGCTTGAGAGCTAACTGGAAGGGAGGGAAGGTGTGCATTCCTGCACCAGGGCCTTTGCATGTACTTGTTACTTTCTCCTGCTGGCCAGCTGGAGTGTCTGGAAAGATCTGCCTTACTCAGGGAAGCCTTCTCACTCGCACTCTGCCCAGTTAGGACCACTGCCCTCTAGAGACCCTATAGAATTACAGAGGAGGCCGGGCGTGATAGCTCACACCTGGAATCCCAGCACTCCAGCCTGGGCGACAGAGTGAGACTCCATCTCAAAAAAAAAAAAGAAAAAAGAATAAACCCCACACACCTTTGCCTTCCCTTTGCTTTCCCTCACCACACCCCCATTCTCCCCGCCTCCCAGCCCCTGGCAACCACTAATCTGCCTCCTGTCTGCGTGGATTTGCCTGTCCTGGACATTTCCTATAAATGGATCATACAATCTGTGGTCCTTTTGTTTGGCCTCTTTCTGTTGGAATAATCTTTTCCAGGGACATCCATGTTGTAGCATGTGTCAGAATTTCCTTTCTTTTTATAGCTGAATAATATCCCATTGTATGGACTGACCATGTTTAGTTGGCCCATCCATCCGCAGATGGACGTTTGGATTGTTTCCACCTTTGGGCTATTGTGAATAATGCTGCTGTGAACGTTTGTTTACAGGTTTTCAATGCAGGCAAATGTTTTCGTTTTTCCTGGGCATAAATTCCAGGAGTGGAATCGCTGGGTCATATGGTAACTCTCTTTAACCGTTTGAGGAACCGTCAGACTGTTCTCCATGGTGGCTGTGCCATTTGACATTCCCACAAGCAGTGGAAATGGGTTCTGGATTTCTCATCCTCACCAACTTTTTTGATTCCAGCCATCCTAGTGGGTGTGAGATGGTGTCTCATTGTGGTTAGGATTTGCATTTCCCTGATGATGTTTTCTTCTAATTTCCCTGATGACTAGAGATGTTTTCATGTAAGTTATTTATTTATTTATTTATTTTTGAGACAGTGTCTCTGTCTGTATCCCAGGCTGGAGTATAGTGGCGCAATCACAGCTCACTGCAGCCTTGACCTGGGCTCAACAACAGATCCACCCCCCACCACACCCTCCCTAGTAGCTGGGACCACAGGTGTGCTCCACCATGCCGGGGTAATTTTTGTATTTTTCGTAAGAGGCAGGGTTTCACCATGCTGCCCAGGCTGGTCTCAATCTCCTAGGCTCAAGTGATCCACACGCCTTGCCCTCCCAAAGTGCTGGGATTACAGGCATGAGCCACTGCACCCAGCCTCATGCAATTTCTTTTTCCTTTTTTTTTTTTGAGACAGTCTTACTCTATGCCCCAGGCTGAAGTGCAGTGCCACAATCTCGGCTCACTGCAACCTCTGTCTCCTGGGTTCAAGTAATTATCTGGCTTCAGCCTCCCTAGTAGCTGGGACTACATACCCACACCACCTTACCTGGCTAATTTTTGTATTTTTGGTAGACAGGGTTTTGCCACGTTGGCCAAGCTGGTCTCGAACTCCTGGCCTCAAGTGATCCGCCTGCCTCAGCCTCCCAAAGTGCTGAGATTACATGAGTGAGCCACTGCGCCTGACCTCATGTAATTTCTTGAGTGGTCATTTAGTCATTATACAAACAGTGATTGAAGGGCATGTGCCCAGCACTGCAGACTCTGCTGCACAGTCAGATGACCCTGCCTTTCTGGCATTTCCATGCCAGTACCCTGAAGAGATACCATATATGTACCTGGTGCTTGCAATAGCTGGCACTCAGAGGTAGCACACTGTGTCCTAGCCACTCTTCCGAGCACTTAAGCATATTCAGTTACTCTTCAAGTACATATGAATAAGTATGCCTTTGCCCCCTCCTCTTTGCAAGGGATAGCGTGTGGCCACCCTGGTCTGCAGCTAGCTTAGCAGATTTGACCACATATTGTAAAAGTGAGTCTGTACCAGGACAGATAGGGTAGAGGGCTCTGTGTGGTTTAGCACCATGGTAGATTCCATCGTGCGGATGTCCCTCCAGCAGGGGAGGTCTTTGGGGCCAGGGCTAGGCCAGCACCCACATTTGCCGCCTGACCCCGTCCCTCCGTCTCTGTCTCTCCATTCCTCCCTGCCCCTCGTGCAGCCGCTGCCATGGCCCAGACACTGCAGATGGAGATCCCGAACTTCGGCAACAGCATCCTGGAGTGCCTCAATGAACAGCGGCTGCAGGGCCTGTACTGTGACGTGTCAGTGGTGGTCAAGGGCCATGCCTTCAAGGCCCACCGGGCCGTGCTTGCTGCCAGCAGCTCCTACTTCCGGGACCTGTTCAACAACAGCCGCAGCGCCGTGGTGGAGCTGCCGGCGGCTGTGCAGCCCCAGTCTTTCCAGCAGATCCTCAGCTTCTGCTACACGGGCCGGCTGAGCATGAACGTGGGCGACCAGTTCCTGCTCATGTACACGGCTGGCTTCCTGCAGATCCAGGAGATCATGGAGAAGGGCACCGAGTTCTTCCTCAAGGTGAGCTCCCCGAGCTGCGACTCCCAGGGCCTGCATGCGGAGGAGGCCCCATCGTCGGAGCCCCAGAGCCCCGTGGCGCAGACATCGGGCTGGCCAGCCTGTAGCACCCCGCTGCCCCTCGTGTCGCGGGTGAAGACGGAGCAGCAGGAGTCGGACTCCGTGCAGTGCATGCCCGTGGCCAAGCGGCTGTGGGACAGTGGCCAGAAGGAGGCTGGGGGCGGCGGCAATGGCAGCCGCAAGATGGCCAAGTTCTCCACGCCGGACCTGGCTGCCAACCGGCCTCACCAGCCCCCGCCACCCCAACAGGCTCCGGTGGTGGCAGCAGCCCAGCCCGCCGTGGCTGCGGGAGCAGGGCAGCCAGCCGGTGGGGTGGCAGCAGCAGGGGGTGTGGTGAGTGGGCCCAGCACGTCGGAGCGGACCAGCCCAGGCACCTCAAGCGCCTACACCAGCGACAGCCCTGGCTCCTACCACAATGAGGAGGACGAGGAGGAGGATGGTGGCGAGGAGGGCATGGATGAGCAGTACCGGCAGATCTGCAACATGTACACCATGTACAGCATGATGAACGTCGGCCAGACAGGTGAGGTGCCGTCCTGTCCCCCATCCCACCAGCCACCCCTGCTCCTCCTGCCACTCGCGTGCCACTCTCTCCCTGCAGCCGAGAAGGTGGAGGCCCTCCCGGAGCAGGTAGCCCCCGAGTCCCGAAATCGCATCCGGGTTCGGCAAGACCTGGCGTCTCTCCCGGCTGAACTTATCAACCAGATTGGGAACCGCTGCCACCCCAAGCTCTACGACGAGGGCGACCCCTCTGAGAAGCTGGAGCTGGTGACAGGTGGGCCGGTCTCGCCCCAGATCTCTCCCCTCCGCAGCTTTGGAGCCGGCTGGCCTGGGCTGGGCTGGGCAGCTGGTTAGGAGCCCCCAGCACCTCAGTTTCCCTATCTGTGCTGTAGTGTTGGTAACAGCCACCCTAAGGGTGGGGGCGTTGGTGAGATGGAGGAGCTGATACAGCAAGGCCTGGCCTCTAGGTTTCTGGCTTAAGGGGTCGGGGCGAGCATTGGCTATTATTGTTTGGCCGCATGGGTAGATTAGCTACTTCCCGGGCTCCTGTGAAGGCCAGCTACTACCTAGGTTAGGAATTTGGACAGTCACAGGGGCGAATGCCTGTAGTCCTAGCTACTCAGGAGGCTGAGGTGGGAGGATCCCTTGAGCCCAGGAGGTCAAGGCTGCAGTGAGCTGTGGTGGCCACTGCACTCCAGCCTGGGTGACAGAGTGAGACTTCATCTCTTAAAAAAAGAAGAAGAAGACTGTGTTTGGTCCTTGGGTCAGAGTGCCTAGGTGTGAGCCTCCACTCCCTGTTGGTCCTGCTGAGCTTGGGCTCATCTCTAGCTGGTGACAGCCAGCGTGCCCACCTCACACAGGCTTGCGGCACGACTGGCCACACAGCAGGCACCCCGAGGATGGTGAAGCAACCCTTTCTGTCCTTTCCTGAGCACTGATGAGGTTGGGGGAGCCCCAAGGAGCCTCCCCTGACTGCCCTTGGTGGGTAGAGATGTAGGGGAGAAGGTCCCTGGGTGAGTTTTCTTGGGACCCAGAGCCCAGCAGGGAGGGCCTGGGGTGTTCTGAGATGAGGCCTGGTATCATGGGGGCTGTGGCGGTTTGGGGGCACCCCAGGCCATCCTCCGGCTTCCTCTCACCAGGCACCAACGTGTACATCACAAGGGCGCAGCTGATGAACTGCCACGTCAGCGCAGGCACGCGGCACAAGGTCCTACTGCGGCGGCTCCTGGCCTCCTTCTTTGACCGGTAAGGCCCTTGCCAGAGCCCCAGGGAGGGGGGTGGGGTTTCCCCATGTCCCCCCCACCACCAACTTGAGCGCTGACTCCCTCCATGTCCCCTGCCCCCAGGAACACGCTGGCCAACAGCTGCGGCACCGGCATCCGCTCTTCTACCAACGATCCCCGTCGGAAGCCCCTGGACAGCCGCGTGCTCCACGCTGTCAAGTGTGAGTGTTGGCCCAGCTGGACGAGGCGTGGGCCCGGGGCACGCAGGTTGACGTTTTTTCCCAGCCTTGGCTCTCAGAGAGGGCTAGAGTTCAGTGTTGAGAAGCATTCTGGGGCTCATTCTAGCCTTGCTGGGAAACCGGCTGTGATTGCTTAGAGATTTCTTCGTGTTTGGGGGATGCACGTGCCGAAGGGAAGCCAGGGAGCCTGTGTCAGGCAGGAGGGTCTCGAACTCAGGGCAGTCTGGGAGGGATGAGGCAGCCAGACAGTGCTAGCCACTCGTCATGGGGGGCATCTAGATTTTCAAGTGACCACTCCCCGTTACTAAATTCTGGCAAATGATCCGAATTTTTCAAGAACACGGGGCCATCCCATCAAAACCAACACATCCCCACACATGGCTGAACTCAGTCCGGTGTAGGGTTGGGTGCACGTAGTGTGGTGTCCTGGCCGCGTGGCCTCACTCGTTTCCCCTTTGAGAGGGAGTCGCAGATGCTGTAGGGGAGCTGGTGAGTAGGCCTTGTGGGAGTCACCTGGCCCCCGTGCCAAGGCCGCACCCACCCTGCCCCACAGACTACTGCCAGAACTTCGCCCCCAACTTCAAGGAGAGCGAGATGAATGCCATCGCGGCCGACATGTGCACCAACGCCCGCCGCGTCGTGCGCAAGAGCTGGATGCCCAAGGTCAAGGTGCTCAAGGCTGAGGATGACGCCTACACCACCTTCATCAGTGAAACGGGCAAGATCGAGCCGGACATGATGGGTGTGGAGCATGGCTTCGAGACCGCCAGCCACGAGGGCGAGGCGGGTCCCTCGGCTGAAGCCCTGCAGTAACCCGCCCAGCCTCCCGCGGGGCCACACACTTCCCCTCCCAACACACACACACACCTGCCATCTTGGTCATGAGCTACTGTCTGTCCCTCCCCAGGACCCGCGGTGGGTGCTGCATGTTCCCGGCCCTCTGCCCCTCCTGTCCTACCCCCTTTCCCCACCGAGAGCTGGGCCGGGAGAGGACCGCAGGGCAGGTGGCGTGAGGTCCGTGTTGCCTTCTTTAACACACACTCGTGCAGTGGGGGAGTTCTGGCTCCCCAACCTAACCCCTAGCCGTCATCTCCACACTCACCAGGCCCACCAGGGGAGGGGGCTGGCCTGGGGGTCTTGGGAAGGCCCCTCCCCAGGCCCTAGGCCACCTCGCGGAAGCCTTCAGCCTCCGCCCCTCACTGCAGCCCCTTGGGACTTGAGGGGGGCCCCAGGGGTTCTCAGGACCCCTCCCACCACCTCCCAGTGCTTCCACGTCTCCAAAAGCGCCTTCCTGTCACCCTCGTCTATCCCTGCGCCTGGGGGCTGGGGTAGGCGAGGCCGTGGGGACTACCCATTTTATAGCTGGGGAAACAGGCTCCGAGAAATTGCACAACCGACCTCAGGTGGCCGGCAGTGGCGTGCCTGGTGCCCTTTCTCCTCTTCCGCCTCTGTTGCTCCCTTTGGTCCTCTGGGGCCTCAGAGAGGCAGGTCCTAAAGGAGGGGGGGTCTCCTGGGGGCAGACCGAGGGTCTCGGAGGATAGAATGAGAGGTTTCCAGGGCTGGCAGAGTGTGTGCGTGTGTGTGTGCAGAAGTTTTGCTTTCAAACAAATGAAGATACAAGAGGCAGTAGGACCTGGGTTGGAACCCAGGGGAGCAGACTGGGGGGCTGTTGCCCCTCCCTCCATCTCCCCACACCCACCCCACCCCACAGCAGGGCTCCTGGCCCCCACCCCCCTGTACATAATTTTTAAAACCTTTTTTTAGCGAATGAAATATTGAAGTATAAGATTCCTTTTATTTTTCAAACCAATGGGGCTGTGTCTATTGTCCCCCTCGGTCCCCAGGGGTGGGGGGCCTTGGAAGGCAGGGCGGTGTTGGGGGGCTGGGGGGCAGGCTGAATGTGTGTGTGTGGGTGTGTATGTGTGGAGTGTGTGTGTGTGTTTCCATGGTAGGACTCCAGTCCCAGCTGCTGCCTAGGCTCCTGTGGGTCCTGTGGGTCCCCCTCCCCTGTTCACCTTCCCCAACAGGGCTATAGTTTAAGGGTGTCCCAGGCCTTGGCTGGTTGGCTGGGCCCAGCTCCTGGGGGCTGGGCAGGGCAGGGCAGGGCCGGGTCCACTTCCATTGTTAGCAGTTGTTTGCAGAATTTTCTCTTTTACCATTCCTCTCTTTTTGTTCTCGCCCAGAGTGGGTGGTTTTTTGTTGTTGTTTTAGATTTTCATGTGAAGGCAGTTTTTTTTCTTTTTTAACTCCCCATCCCCCTATGCAGGGTGTCATTTCACCATCCACATCCACATCCTTGTTTGTGTTTCTGGCCCTCGTCCTCCCTGCCTCTCATCTGCCTCCCCAACCCCCACCTACCCACTGCTCCCAGGGGTCTCTCCAGGGGCAGGGGGATTTTAGGAGATGGGGTGGGGGCCAGCCCCTACTGGACCCTTGTAATCTGATTTGGAGACCATCCCCCCGATGGGACCAGCAGCCCCGCCGGCCCACTGGGGGTCCCTCTTAACATCTGCTTGTTGCGGGGGTCAGTCATGAGTGCCAGGGTCTGGCTGGGGTCTTGGCACTGTCTTCCTGGCCGGGGCCTGAAACCTTAGCCCCCAGCCGGCCTGAGTGGGGGATATTGACACCCCCAGCTAAAGCACAAGCACCTTAGTCACCCCTCCTCTCCCCCCGCCTCCTGGCCTGCCACCCCACCCCAGCGTTTACCCCAAAGCACAATGCCCTGGTCACTTGGCAAGCGGCTGGGCCTGACGGAGGCCGAGGGGCAGACCGGGGCTCCAGCCAGACCCCTGAAAGGAAGGTGCTCCCCCACTTCCAGGCCTTAGTTGGGGTGGGGGGGCCAGCTCCTGGGCCTGGGGTCTTCTCACCCCCACCCCCTTGTCCTGGGTAGGCCCCTGCCTGCCCCTCTCTGCCTTTTCCTCGGGTGTCCCTCCTCGAGCCCCTGTGGCATTGGCTTGGGTGGCAGAGCCCACTTGTTTCAGGGACCCCAGGAGGTGCCCCCTGGCTCCCAGGACTGTGTGTGGGTCCGGGGGGTGGGGGGGTGGGGAGAAGGGTCGGGCAGGGGGTGCAGGGGAGGAAACTCCTCACCAGGAGAGCCAAAGACAGGGTTGTGCCTTACCCCAGGAGCCACCCCTGTACCCCCCTTGCCCTGCCATTCACCCTCCACCCCTACCCCTGGGCGGCCTGCTGCTTTTTCCTTCTCTTCCTCCCCTGCCCTGAGCTGCATGGTTCCCCCACCCTGGGCAGCCAGGAAGGAATCTGAATGGAGAATCACCAACCACCAGAGAAAAAAGACTGTGGGGCCCTCCCCTGCCAACTCCCCTTCCCTGGCCGCCCACTCAACCCACACCTCTTTCACGCAGGACAGGCTGCCCACCCTGTCCACGTGAAGTGCCAACGCCCTCCCCACCCTGGGCCGAGCCCCCACCCCTCCCTGGGCCCCCAAGTGAGATTGCACATTTAACTACTGTAAGGAGAGGAGCGGCGTTGGCAAATGTGAACCATGAGAATATCAGTGATACTGATGAGAATAAACTAAACGCCTTTGTAACAGC

**sh-FOXP4-AS1 (5’-3’)**

Top Strand

CACCGCAATTATCCGGACAAATTAGCGAACTAATTTGTCCGGATAATTGC

Bottom Strand

AAAAGCAATTATCCGGACAAATTAGTTCGCTAATTTGTCCGGATAATTGC

**miR-423-5p mimics (5’-3’)**

UGAGGGGCAGAGAGCGAGACUUU

**miR-423-5p inhibitor (5’-3’)**

AAAGUCUCGCUCUCUGCCCCUCA

**sh-NACC1 (5’-3’)**

Top Strand

CACCGCTAATTGCAAGGCTGTTACCCGAAGGTAACAGCCTTGCAATTAGC

Bottom Strand

AAAAGCTAATTGCAAGGCTGTTACCTTCGGGTAACAGCCTTGCAATTAGC
